# Adhesive Photoinitiator Constructs Polymer Jackets on Enzymes: Direct, Release‐Free Cytosolic Delivery

**DOI:** 10.1002/anie.202524301

**Published:** 2026-01-30

**Authors:** Shuran He, Soumen Ghosh, Kou Okuro

**Affiliations:** ^1^ Department of Chemistry The University of Hong Kong Hong Kong SAR P.R. China; ^2^ State Key Laboratory of Synthetic Chemistry The University of Hong Kong Hong Kong SAR P.R. China

**Keywords:** drug delivery, enzyme protection, grafting‐from polymerization, membrane translocation, supramolecular chemistry

## Abstract

Enzyme therapeutics require both catalytic activity and efficient cytosolic delivery—yet protective encapsulation typically compromises enzymatic function, while achieving cellular uptake without lysosomal degradation remains challenging. We address this with a rationally designed supramolecular adhesive photoinitiator (^Gu^CD⊃BP‐SH) that unifies surface adhesion, radical initiation, and membrane translocation within a single host‐guest architecture. Guanidinium (Gu^+^) motifs on a cyclodextrin scaffold (^Gu^CD) enable non‐covalent adhesion to protein surfaces at carboxylate‐rich regions; the cyclodextrin cavity hosts a thiol‐benzophenone guest (BP‐SH) whose photoactivation (365 nm, 60 mW cm^−2^ for 30 min) initiates localized grafting‐from polymerization, constructing a semi‐permeable polymer jacket. Applied to *β*‐galactosidase, this yields sub‐100 nm multi‐enzyme nanoassemblies (containing ∼10 enzymes per particle) retaining ∼30% catalytic activity with exceptional proteolytic resistance: 86% activity retained versus 25% for unprotected enzyme after Proteinase K challenge. The incorporated Gu^+^ motifs enable efficient, energy‐independent cytosolic delivery via membrane translocation, with 91% of cells showing catalytic activity compared to 5% with non‐jacketed enzyme. This modular strategy confers protection and cell‐penetrating capability onto native biomacromolecules while maintaining catalytic function, eliminating the need for enzyme release—a persistent bottleneck in therapeutic delivery.

## Graphical abstract

A supramolecular adhesive photoinitiator, ^Gu^CD⊃BP‐SH, overcomes a central dilemma in enzyme therapeutics. The initiator, featuring a guanidinium‐modified cyclodextrin (^Gu^CD) host, triggers formation of a semi‐permeable polymer jacket directly on an enzyme. This jacket uniquely provides proteolytic resistance and enables direct cytosolic delivery, all while preserving significant catalytic function, thus unifying protection and substrate access.

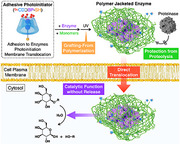

Intracellular enzyme delivery is a promising approach for treating diseases intractable to small‐molecule therapies [1, 2]. Among various strategies including nanocarrier‐mediated endocytosis, direct translocation across the cell membrane offers immediate cytosolic access without lysosomal degradation [3, 4, 5, 6, 7, 8, 9] yet remains a formidable challenge [10, 11]. For instance, covalent conjugation of cell‐penetrating peptides (CPPs) [12, 13, 14, 15] can irreversibly denature the enzyme [16, 17]. To address this limitation, we previously developed “molecular glues”—linear or dendritic polymers with multiple guanidinium (Gu^+^) pendants that adhere to proteins via multivalent salt‐bridge interactions with surface carboxylates [18, 19]. These multivalent interactions enable binding to cell surfaces, facilitating direct cytosolic delivery of the enzyme without harsh covalent modifications [20, 21, 22, 23, 24]. This success, however, exposed a fundamental dilemma common to enzyme‐based hybrid materials designed for therapy. Strategies such as polymer wrapping or encapsulation typically enhance stability [25, 26, 27], yet the layer that enables cellular uptake or protection simultaneously compromises enzymatic activity through steric hindrance (Figure 1a,b, and d). While this necessitates subsequent enzyme release, achieving the spatiotemporal control required for this process remains a major therapeutic barrier [28, 29, 30, 31, 32, 33, 34].

**FIGURE 1:**
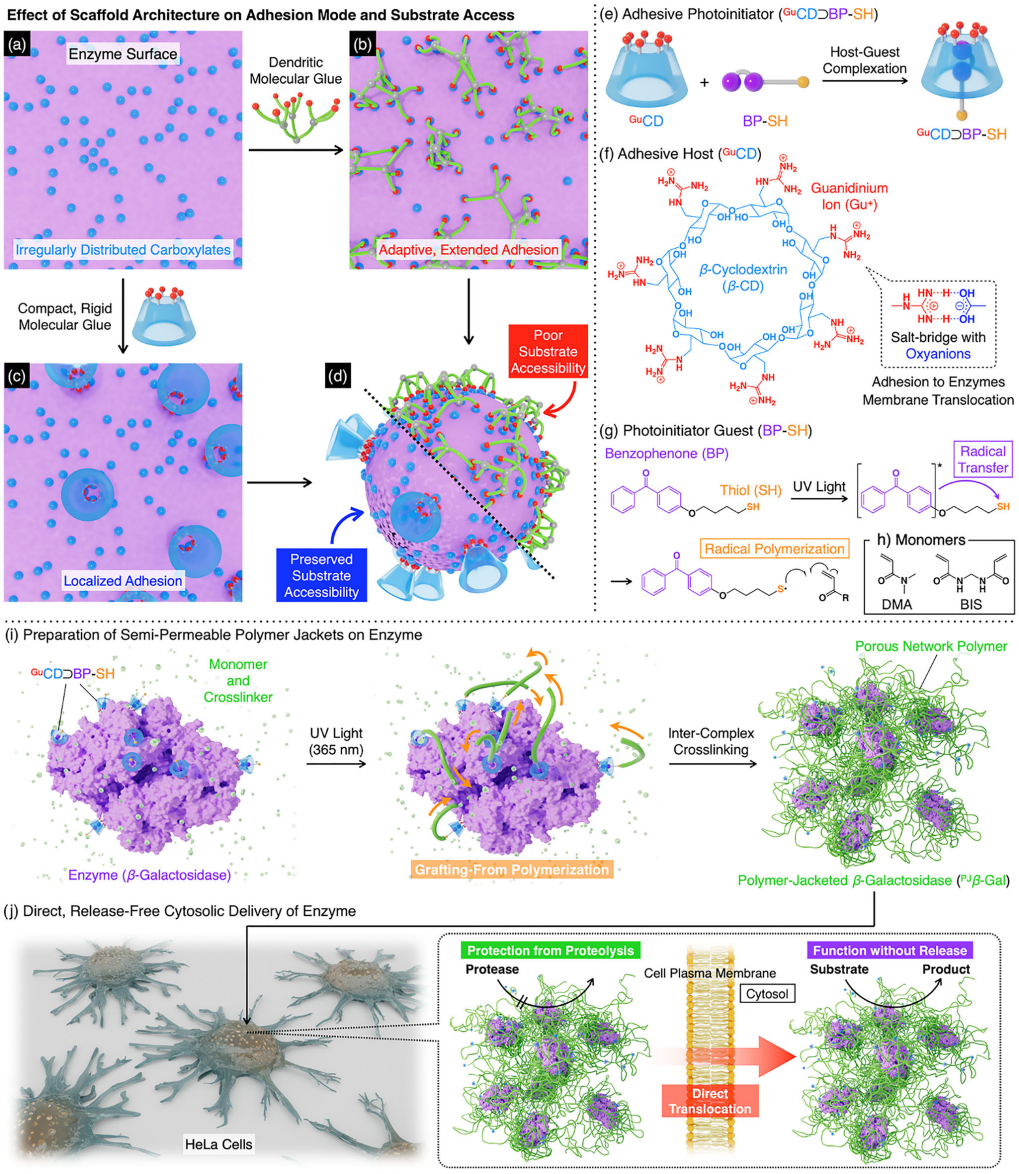
(a–d) Schematic illustration of the scaffold design concept regarding adhesion modes on an enzyme surface with irregularly distributed carboxylates (a). Flexible dendritic scaffolds result in extended adhesion (b), hindering substrate accessibility (d, top). In contrast, compact, rigid scaffolds are designed to enable localized adhesion preferentially at carboxylate‐rich regions (c), thereby preserving substrate accessibility (d, bottom). (e–h) Components for polymer jacketing. A supramolecular adhesive photoinitiator, ^Gu^CD⊃BP‐SH (e), comprises a guanidinium (Gu^+^)‐modified *β*‐cyclodextrin host (^Gu^CD, f), and a benzophenone‐thiol guest initiator (BP‐SH, g). (h) Monomer (DMA) and crosslinker (BIS) for polymerization. (i) Schematic of polymer jacket formation on *β*‐galactosidase (*β*‐Gal). Upon UV irradiation, noncovalently bound ^Gu^CD⊃BP‐SH triggers localized grafting‐from polymerization, yielding polymer‐jacketed enzyme (^PJ^ *β*‐Gal). (j) The jacket confers proteolytic resistance, enables membrane translocation, and preserves catalytic activity without enzyme release.

Resolving this dilemma requires a departure from conventional encapsulation strategies. We designed a supramolecular adhesive photoinitiator, ^Gu^CD⊃BP‐SH (Figure 1e), that unifies three essential functions: surface adhesion, radical generation, and membrane penetration. Central to this design is a Gu^+^‐functionalized *β*‐cyclodextrin (^Gu^CD, Figure 1f) host [35, 36, 37] that provides surface adhesion and membrane translocation, while encapsulating a thiol‐benzophenone (BP‐SH, Figure 1g) guest as a spatially confined photoinitiator. Flexible polymers such as linear chains or dendrimers adaptively bind to irregularly distributed carboxylates on protein surfaces, leading to broad coverage (Figure 1b and d). In contrast, the rigid cyclodextrin scaffold of ^Gu^CD precludes such conformational adjustments, enforcing Gu^+^‐mediated salt‐bridge adhesion at carboxylate‐rich regions where the geometric arrangement matches the Gu^+^ motifs (Figure 1c and d). Presenting the Gu^+^ motifs on this compact, rigid scaffold minimizes surface coverage while maintaining stable anchoring. Upon UV irradiation, intramolecular radical transfer from photoexcited benzophenone to the thiol (Figure 1g) [38, 39, 40] initiates localized grafting‐from polymerization (Figure 1i). This process yields a porous polymer jacket that covalently incorporates the ^Gu^CD host and possesses membrane‐penetrating ability, eliminating the need for subsequent release mechanisms (Figure 1j). In addition to enabling direct membrane translocation, the semi‐permeable jacket architecture sterically excludes proteases while permitting small‐molecule diffusion, thereby conferring proteolytic stability.

Formation of the target 1:1 inclusion complex, ^Gu^CD⊃BP‐SH, was confirmed spectroscopically (Figures S1–S15). ^1^H NMR spectroscopy in D_2_O (Figure S15) revealed the solubilization of the otherwise insoluble BP‐SH guest upon addition of the ^Gu^CD host, consistent with encapsulation. Direct evidence came from 2D ROESY and NOESY spectra, which displayed clear cross‐peaks between the host protons (C3/C5‐*H*, *δ* 3.75 ppm) and the guest aromatic protons (*H*_a_, *δ* 7.77 ppm; *H*_e_, *δ* 7.01 ppm), demonstrating inclusion complex formation (Figure 2a and b). UV–vis spectroscopy further corroborated complex formation, showing the characteristic benzophenone absorption (∼290 nm) in water (Figures 2c and S17). The complex was kinetically stable, showing no appreciable dissociation, consistent with entrapment of the hydrophobic guest within the host cavity. A reference photoinitiator lacking the adhesive Gu^+^ groups (CD⊃BP‐SH) were prepared for control studies (Figure S16).

**FIGURE 2:**
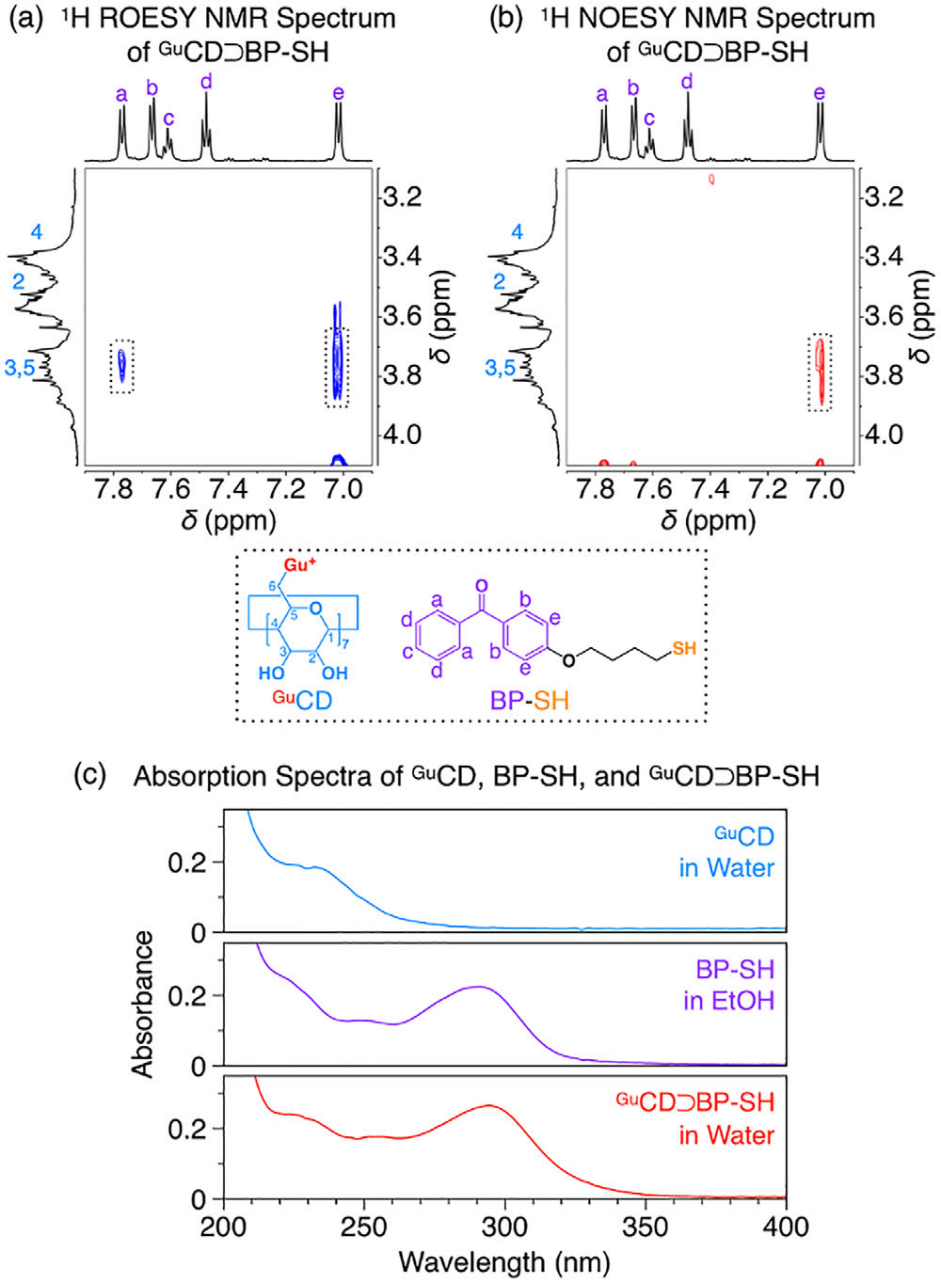
(a, b) ^1^H ROESY (a) and ^1^H NOESY (b) NMR spectra of ^Gu^CD⊃BP‐SH (5 mg mL^−1^) in D_2_O at 25 °C. (c) Absorption spectra (1 mg mL^−1^) of ^Gu^CD in water, BP‐SH in ethanol (EtOH), and ^Gu^CD⊃BP‐SH in water.

Having established the adhesive photoinitiator, we constructed the polymer jacket on *β*‐galactosidase (*β*‐Gal), a widely used model enzyme with established roles as a reporter and therapeutic [41, 42], bearing irregularly distributed surface carboxylates (Figure S22). For visualization, the jacket was grown in situ on FITC‐labeled *β*‐Gal (*β*‐Gal^FITC^). *β*‐Gal^FITC^ (2 *µ*M) was mixed with ^Gu^CD⊃BP‐SH (60 *µ*M), followed by the addition of *N*,*N*‐dimethylacrylamide (DMA, 50 mM; Figure 1h), selected to ensure stable enzyme retention without denaturation [29], and *N*,*N'*‐methylene bis(acrylamide) (BIS, 50 mM; Figure 1h). The mixture was then subjected to UV irradiation (365 nm, 60 mW cm^−2^) for 30 min, and the resulting polymer‐jacketed enzyme (^PJ^ *β*‐Gal^FITC^) was isolated by dialysis. Dynamic light scattering (DLS) of ^PJ^ *β*‐Gal^FITC^ revealed a significant increase in the average hydrodynamic diameter (*D*_h_) to 84 ± 20 nm, compared to 7.2 ± 2.5 nm for the native enzyme (Figure 3a) [43]. Transmission electron microscopy (TEM) confirmed this size increase, revealing spherical particles with diameters of 50–100 nm (Figure 3b). The substantial size increase suggested co‐assembly of multiple enzyme molecules, while the shift in zeta potential (*ζ*) to ‐5.1 mV (Figure 3c) and the absence of free *β*‐Gal^FITC^ bands in SDS‐PAGE (Figure S21) confirmed effective polymer coating. Circular dichroism spectroscopy indicated that the polymerization process did not significantly alter the secondary structure of the enzyme (Figure S20). The multi‐enzyme composition was confirmed by combined fluorescence analysis (Figure S23) and nanoparticle tracking (NTA, Figure S24) [43], which yielded an average of 9.8 *β*‐Gal^FITC^ molecules per particle (Figure 3d). This finding indicates an inter‐complex crosslinking mechanism that drives the controlled co‐assembly of multiple enzymes into discrete nanostructures. Crucially, control experiments using the non‐adhesive analog CD⊃BP‐SH yielded no polymer jacket, with *D*_h_ and *ζ* remaining essentially unchanged (Figure S18). Similarly, the addition of sodium tripolyphosphate (TPP), a competitive anion for Gu^+^, inhibited the jacket formation (Figure S19). These results confirm that Gu^+^‐mediated surface adhesion is essential for polymerization, by localizing photogenerated radicals at sufficient concentrations to initiate the reaction. Furthermore, the properties of these nanostructures were readily tunable; for instance, increasing monomer concentrations yielded larger particles, while adjusting the initiator‐to‐enzyme ratio systematically controlled the surface charge (Table S1).

**FIGURE 3:**
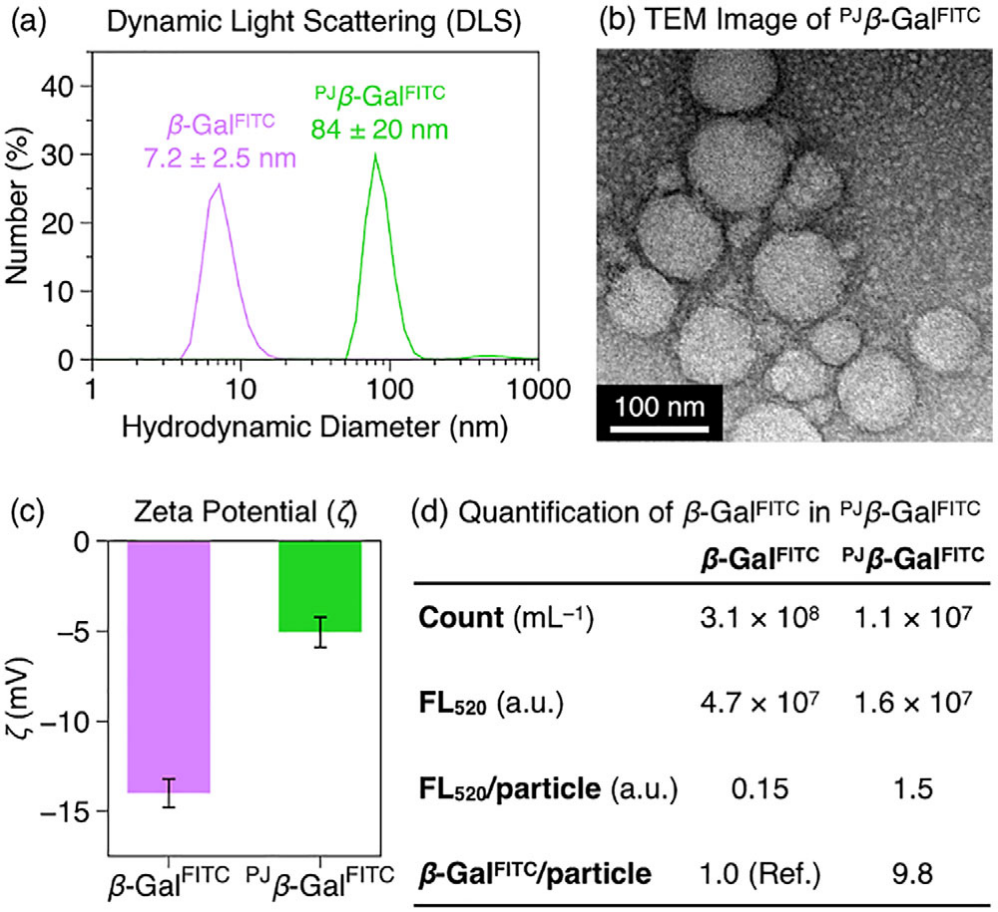
(a) Dynamic light scattering (DLS) profiles of *β*‐Gal^FITC^ (5 mg mL^−1^) and ^PJ^ *β*‐Gal^FITC^ (5 mg mL^−1^) in HEPES buffer (100 mM, pH 7.3). (b) TEM image of ^PJ^ *β*‐Gal^FITC^ after negative staining with 2% uranyl acetate. (c) Zeta potentials of *β*‐Gal^FITC^ (5 mg mL^−1^) and ^PJ^ *β*‐Gal^FITC^ (5 mg mL^−1^) in HEPES buffer (100 mM, pH 7.3). (d) Summary of the analysis to quantify the number of *β*‐Gal^FITC^ molecules per ^PJ^ *β*‐Gal^FITC^ particle, based on particle concentrations measured by nanoparticle tracking analysis (NTA) and total fluorescence intensity at 520 nm (FL_520_; *λ*_ex_ = 488 nm). Data in (a, c) are presented as the mean ± SD (*n* = 3).

The polymer jacket preserved substantial enzymatic activity, a critical feature that distinguishes it from conventional encapsulation methods. Kinetic analysis of ^PJ^ *β*‐Gal^FITC^ using the chromogenic substrate *o*‐nitrophenyl‐*β*‐galactoside (ONPG, Figure 4a) [44] revealed a catalytic efficiency (*k*_cat_/*K*_m_) of 3.11 × 10^3^ M^−1^ s^−1^, corresponding to ∼30% of that of the native enzyme (Figures 4b–d, S26, and Table S2) [45]. This moderate reduction was attributed to a ∼2.2‐fold increase in the Michaelis constant (*K*_m_) from 1.04 to 2.29 mM, indicating reduced substrate affinity due to the porous network. Additionally, the catalytic rate (*k*_cat_) decreased by ∼30% from 10.3 to 7.1 s^−1^, potentially reflecting mild non‐competitive inhibition [29]. This notable retention of activity directly validates our design hypothesis: the resulting semi‐permeability of the crosslinked network enables substrate turnover without requiring a release trigger. Importantly, the jacket conferred exceptional proteolytic stability. When challenged with Proteinase K (ProK; 1:1 molar ratio, replenished every 30 min) [46, 47], unprotected *β*‐Gal^FITC^ retained only 25% of its activity after 3.5 h, whereas ^PJ^ *β*‐Gal^FITC^ maintained 86% (Figure 4e). This striking protection stems from the semi‐permeability of the jacket, which permits substrate access while sterically excluding the protease—a decisive advantage for therapeutic applications requiring prolonged in vivo performance [48, 49, 50].

**FIGURE 4:**
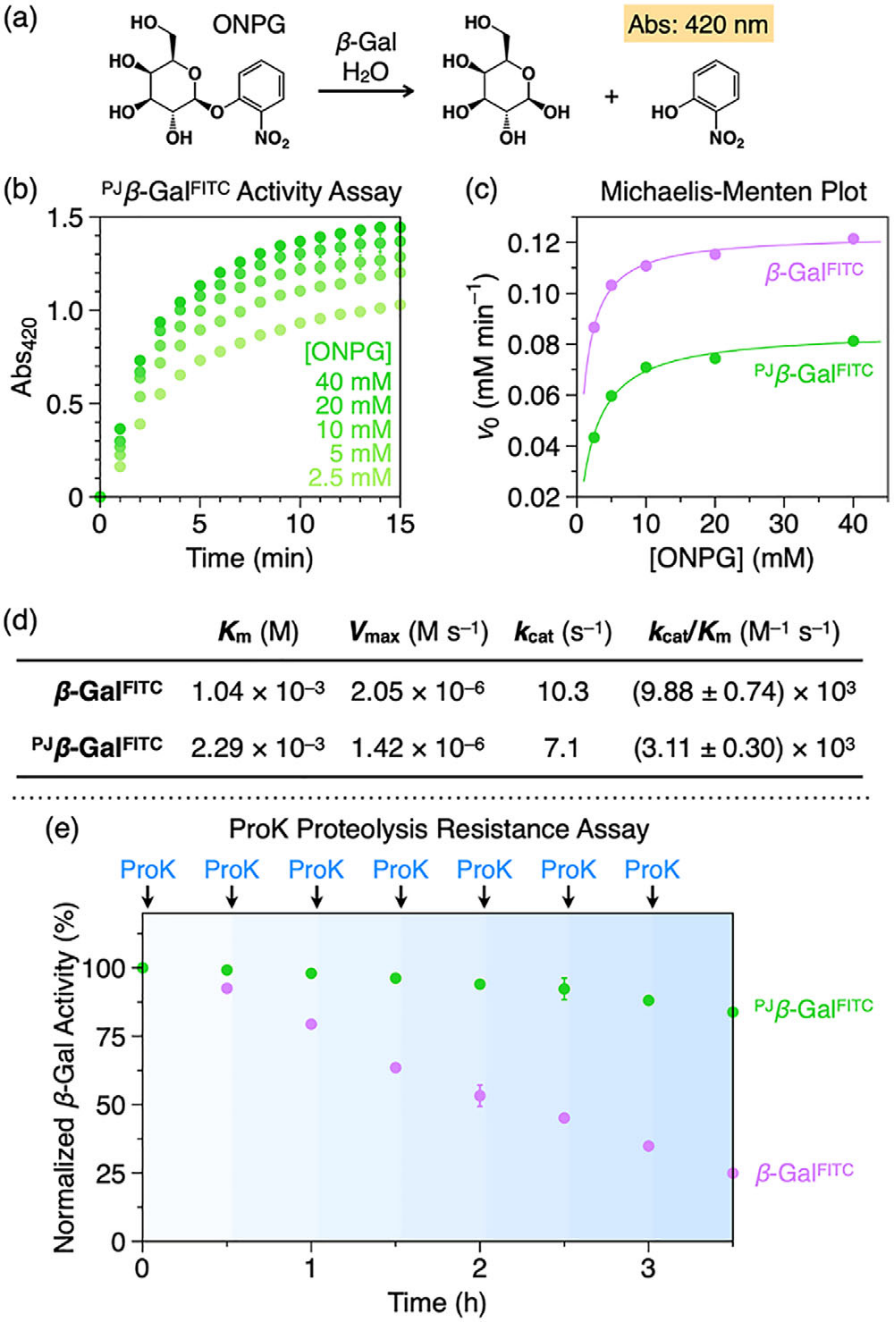
(a) Scheme for the hydrolysis of *o*‐nitrophenyl‐*β*‐D‐galactopyranoside (ONPG) catalyzed by *β*‐Gal. (b) Changes in absorbance at 420 nm (Abs_420_) for 0–15 min at 37 °C in HEPES buffer (100 mM, pH 7.3) containing ^PJ^ *β*‐Gal^FITC^ ([*β*‐Gal] = 0.2 *µ*M) and ONPG at different concentrations (2.5–40 mM). (c) Michaelis‐Menten plots for *β*‐Gal^FITC^ and ^PJ^ *β*‐Gal^FITC^, derived from the initial rates of Abs_420_ change (data for *β*‐Gal^FITC^ are shown in Figure S25). (d) Kinetic parameters for *β*‐Gal^FITC^ and ^PJ^ *β*‐Gal^FITC^. The values were determined by fitting the model to the averaged data from three independent experiments. Errors for *k*_cat_/*K*_m_ represent the standard error of the fit. (e) Normalized activities of *β*‐Gal^FITC^ and ^PJ^ *β*‐Gal^FITC^ over time with repeated additions of Proteinase K (ProK; indicated by arrows) in HEPES buffer (100 mM, pH 7.3) at 37 °C. Data in (b, e) are presented as the mean ± SD (*n* = 3).

After confirming low cytotoxicity at the working concentration (Figure S27a), the ability of the polymer jacket to mediate cellular entry was investigated in human cervical carcinoma HeLa cells. Cells incubated with ^PJ^ *β*‐Gal^FITC^ exhibited intense FITC fluorescence distributed throughout the cytosol and nucleus; in sharp contrast, non‐jacketed *β*‐Gal^FITC^ showed negligible signal (Figure 5a). The observed nuclear localization is consistent with the function of the Gu^+^ groups, which can mimic arginine‐rich nuclear localization signals (NLSs) [51, 52, 53]. Minimal colocalization with the lysosomal marker LysoTracker Red indicates that the jacket facilitates direct membrane translocation, largely bypassing the endolysosomal pathway. Flow cytometry quantified this uptake (Figure 5b): ^PJ^ *β*‐Gal^FITC^‐treated cells exhibited intense fluorescence (mean intensity: 8.3 × 10^5^), whereas cells treated with the non‐jacketed enzyme showed negligible signal (1.3 × 10^3^), comparable to untreated cells (1.1 × 10^3^) [43]. These results provide compelling evidence that the polymer jacket is essential for efficient cytosolic and nuclear delivery of the enzyme. This capability for cellular entry was also confirmed in mouse mammary carcinoma 4T1 cells (Figures S27b and S28).

**FIGURE 5:**
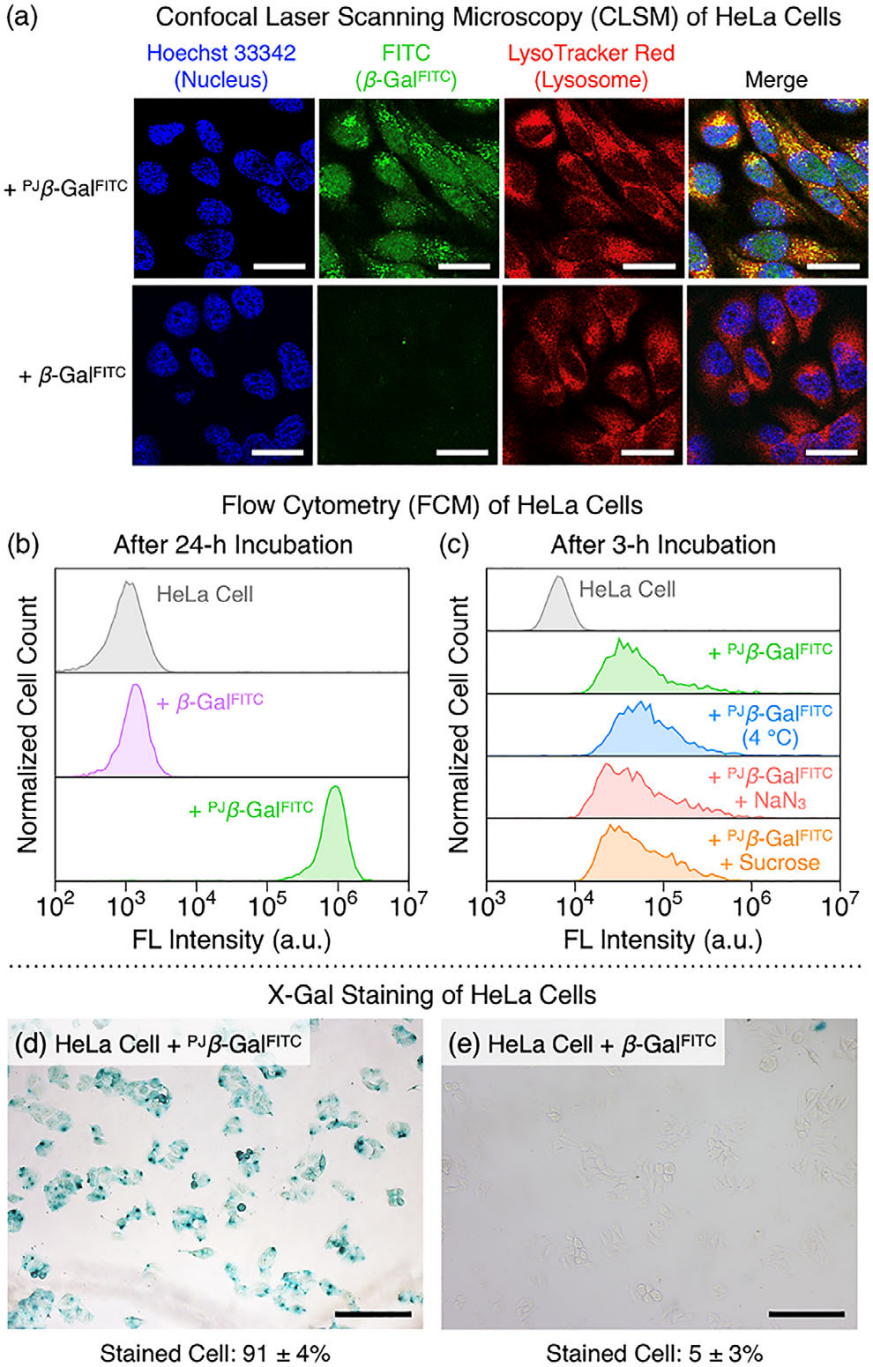
(a) Confocal laser scanning microscopy (CLSM) of HeLa cells prepared by a 24 h incubation with *β*‐Gal^FITC^ (0.1 mg mL^−1^) or ^PJ^ *β*‐Gal^FITC^ (0.1 mg mL^−1^), followed by staining with Hoechst 33342 and LysoTracker Red. Images display fluorescence from Hoechst 33342 (blue; *λ*_ex_ = 405 nm), FITC (green; *λ*_ex_ = 488 nm), LysoTracker Red (red; *λ*_ex_ = 561 nm), and their merged view. (b, c) Flow cytometry (FCM) histograms (*λ*_ex_ = 488 nm) of HeLa cells: (b) after a 24 h incubation with *β*‐Gal^FITC^ (0.1 mg mL^−1^) or ^PJ^ *β*‐Gal^FITC^ (0.1 mg mL^−1^), and (c) after a 3 h incubation with ^PJ^ *β*‐Gal^FITC^ (0.1 mg mL^−1^) with or without endocytosis inhibitors (4 °C, 10 mM NaN_3_, or 400 mM sucrose). Data represent the distribution of cell populations from a single experiment. (d, e) Bright‐field images of HeLa cells stained with X‐Gal after a 24 h pre‐incubation with: (d) ^PJ^ *β*‐Gal^FITC^ (0.25 mg mL^−1^) and (e) *β*‐Gal^FITC^ (0.25 mg mL^−1^). Percentages of stained cells are represented as the mean ± SD (*n* = 3). Statistical analysis was performed using Student's t‐test (*p* < 0.001). Scale bars: 25 *µ*m (a) and 200 *µ*m (d, e).

To elucidate the uptake mechanism, cellular entry was quantified under conditions that block endocytosis by different mechanisms: energy depletion (4 °C or 10 mM NaN_3_) and clathrin pathway disruption (400 mM hypertonic sucrose) with a 1 h pre‐incubation followed by a 3 h incubation with ^PJ^ *β*‐Gal^FITC^ [54, 55, 56, 57, 58]. Flow cytometry analysis revealed that ^PJ^ *β*‐Gal^FITC^ uptake was largely unaffected by these conditions, demonstrating its independence from endocytic pathways (Figure 5c) [43]. This energy‐independent uptake is particularly striking given the substantial particle size (50–100 nm), which far exceeds conventional size limitations for direct membrane translocation [10, 11]. Indeed, the mean fluorescence intensity values were virtually identical to those at 37 °C without inhibitors. Furthermore, the uptake was significantly inhibited by TPP in a concentration‐dependent manner (Figure S29), most likely due to the competitive binding of TPP to Gu^+^ groups. These results indicate that cellular entry occurs predominantly via energy‐independent direct membrane translocation, mediated by the incorporated Gu^+^ motifs from the ^Gu^CD host. Finally, the catalytic competence of the internalized enzyme was confirmed by intracellular X‐Gal staining [59]. Cells treated with ^PJ^ *β*‐Gal^FITC^ exhibited intense blue coloration (91 ± 4% positive, Figure 5d), whereas those treated with the non‐jacketed enzyme showed only negligible staining (5 ± 3%, Figure 5e). This dramatic contrast demonstrates that our polymer jacket approach successfully addresses the fundamental dilemma between efficient cellular delivery and functional preservation.

In conclusion, we have established a supramolecular grafting‐from strategy that reconciles the protection‐function dilemma in enzyme therapeutics through site‐selective polymer jacket formation. The adhesive photoinitiator ^Gu^CD⊃BP‐SH, featuring Gu^+^ groups on a compact, rigid cyclodextrin scaffold, enables salt‐bridge‐mediated surface adhesion and subsequent in situ growth of a semi‐permeable polymer jacket that simultaneously confers proteolytic resistance, facilitates direct cytosolic delivery, and preserves catalytic function without requiring enzyme release. This work establishes a versatile strategy for biomacromolecular engineering, offering a modular approach with broad applications across therapeutic, catalytic, and sensing fields.

## Conflicts of Interest

The authors declare no conflict of interest.

## Supporting information




**Supporting File 1**: The authors have cited additional references within the Supporting Information [60].

## Data Availability

The data that support the findings of this study are available in the Supporting Information of this article.
