# Supplementary material for: Adhesive Photoinitiator Constructs Polymer Jackets on Enzymes: Direct, Release‐Free Cytosolic Delivery
Source: Angew Chem Int Ed Engl. 2026 Jan 30;65(11):e24301. doi: 10.1002/anie.202524301 (PMC12970516; doi:10.1002/anie.202524301)
Supplement: Supplementary file 1 — Supporting File 1: The authors have cited additional references within the Supporting Information [60]. [file ANIE-65-e24301-s001.pdf]

Supporting Information

Adhesive Photoinitiator Constructs Polymer Jackets on Enzymes: Direct, Release-Free Cytosolic Delivery

Shuran He,[a] Soumen Ghosh,[a,b] and Kou Okuro*[a,b]

[a]*Department of Chemistry, The University of Hong Kong,*

Pokfulam Road, Hong Kong SAR, P. R. China

[b]*State Key Laboratory of Synthetic Chemistry, The University of Hong Kong,*

Pokfulam Road, Hong Kong SAR, P. R. China

Table of Contents

1. **Experimental Protocols** S2
2. **Synthesis and Characterization** S7
3. **Structural Analysis of *β*-Galactosidase Surface** S24
4. **Fluorescence Spectroscopy** S25
5. **Nanoparticle Tracking Analysis (NTA)** S26
6. **Enzymatic Activity Assay** S27
7. **Cell Viability Assay** S28
8. **Confocal Laser Scanning Microscopy (CLSM)** S29
9. **Flow Cytometry** S30

1. Experimental Protocols

1-1. General

^{1}H and ^{13}C NMR spectra were recorded on Bruker type AVANCE III spectrometers operating at 400, 500, or 600 MHz, where chemical shifts for ^{1}H NMR spectroscopy were determined with respect to non-deuterated solvent residues; $CHCl_3$ (δ 7.26), DMSO (δ 2.50), and D_2O (δ 4.79), and those for ^{13}C NMR spectroscopy were determined with respect to $CHCl_3$ (δ 77.2) and DMSO (δ 39.5). Electrospray ionization mass (ESI-MS) spectrometry was performed on a Bruker Daltonics Impact II QTOF spectrometer. Normal-phase column chromatography was performed using DAVISIL silica gel (particle size 40–63 μm). Electronic absorption and fluorescence spectra were recorded on an Implen model NP80 NanoPhotometer and a Molecular Devices model SpectraMax iD5 multi-mode microplate reader, respectively. Circular dichroism (CD) spectra were recorded on a JASCO model J-815 spectropolarimeter. Photoirradiation was carried out with a Zhongshan Jigu Lighting Electric Factory LED light source model UVGO 3535-3W (λ = 365 nm). Ultrasonication was performed using a Sonics ultrasonic processor model VCX130. Dynamic light scattering (DLS) measurements were carried out using a Malvern Zetasizer Pro equipped with a He-Ne (633 nm) laser. Transmission electron microscopy (TEM) was conducted using an FEI Tecnai G2 20 scanning TEM, with samples negatively stained prior to imaging. Confocal laser scanning microscopy was performed on a Leica model TCS-SP8. Flow cytometric analyses were performed on a Beckman Coulter CytoFLEX flow cytometer. Quantification of nanoparticles was carried out using a Particle Metrix model PMX 120-Z nanoparticle tracking analyzer. Bright-field optical images for X-Gal staining were acquired using a Nikon ECLIPSE LV100POL optical microscope operated in standard transmitted light mode.

Unless otherwise noted, reagents and solvents were used as received from commercial sources without further purification. Tris-HCl buffer (1.0 M, pH 7.4), β-galactosidase (>8.0 units mg^{-1}), ortho-nitrophenyl-β-D-galactopyranoside (ONPG), Enhanced Cell Counting Kit-8, Trypsin-EDTA solution, LysoTracker Red, In Situ β-galactosidase Staining Kit, and HeLa cells were purchased from Beyotime Biotechnology. 4T1 cells were purchased from ATCC. *N*,*N*-Dimethylacrylamide (DMA), *N*,*N*'-methylenebis(acrylamide) (BIS), 3-buten-1-ol, and dimethoxy-2-phenylacetophenone were purchased from Bide Chemical. Triphenylphosphine

was purchased from TCI. Hydrogen chloride (4 mol L^{-1} in 1,4-dioxane) and sodium azide were purchased from Sigma Aldrich. Sucrose was purchased from Amethyst. Tris(2-carboxyethyl) phosphine hydrochloride was purchased from Energy Chemical. Sodium tripolyphosphate (TPP) was purchased from Macklin. Dulbecco's Modified Eagle Medium (DMEM), Dulbecco's Phosphate-Buffered Saline (D-PBS), and fetal bovine serum (FBS) were purchased from Thermo Fisher Scientific. Dichloromethane, methanol, and dimethylformamide were pre-dried using activated molecular sieves before use.

1-2. Preparation of ^{Gu}CD⊃BP-SH and CD⊃BP-SH

An ethanol (EtOH, 3 mL) solution of BP-SH (0.6 mmol) was added to an aqueous (1.5 mL) solution of ^{Gu}CD (0.1 mmol), and the mixture was subjected to pulsed ultrasonication for 24 h with cycles of 50 s on and 10 s off. The resulting mixture was evaporated to dryness under reduced pressure, resuspended in water, filtered off from an insoluble fraction, and then lyophilized to afford ^{Gu}CD⊃BP-SH.

Likewise, CD⊃BP-SH was prepared using CD (0.1 mmol) instead of ^{Gu}CD (0.1 mmol) under otherwise identical conditions.

1-3. Preparation of FITC-labeled *β*-Galactosidase (*β*-Gal^{FITC})

A DMSO solution of fluorescein isothiocyanate (FITC) was added to a HEPES buffer (100 mM, pH 7.3) solution of *β*-galactosidase (*β*-Gal) to give final concentrations of 2 mg mL^{-1} and 1 μg mL^{-1}, respectively. The mixture was stirred at room temperature for 5 h in the dark. The reaction mixture was dialyzed against water (molecular weight cut-off: 8–14 kDa), chromatographed on a desalting column (Cytiva, PD MidiTrap G-25), and then lyophilized to afford *β*-Gal^{FITC}.

1-4. Preparation of ^{PJ}*β*-Gal^{FITC}

Typically, DMA (50 mM) and BIS (50 mM) were added to a HEPES buffer (100 mM, pH

7.3) solution containing β-Gal^{FITC} (2 μM) and ^{Gu}CD⊃BP-SH (60 μM), and the mixture was purged with argon gas for 10 min. The resulting mixture was exposed to UV light at 365 nm for 30 min, followed by dialysis against HEPES buffer (molecular weight cut-off: 8–12 kDa) to yield $^{PJ}\beta$-Gal^{FITC}.

A reference sample was prepared using CD⊃BP-SH (60 μM) instead of ^{Gu}CD⊃BP-SH (60 μM) under otherwise identical conditions. Another reference sample was prepared using ^{Gu}CD⊃BP-SH (60 μM) in the presence of sodium tripolyphosphate (TPP, 280 μM) under otherwise identical conditions.

1-5. Enzymatic Activity Assay

Typically, a HEPES (100 mM, pH 7.3) buffer solution of β-Gal^{FITC} (0.2 μM) or $^{PJ}\beta$-Gal^{FITC} ([β-Gal^{FITC}] = 0.2 μM) was mixed with ONPG (2.5–40 mM) and subjected to absorbance (420 nm) measurement at 37 °C. The initial reaction rates, determined from the linear increase in absorbance at 420 nm, were used to represent the enzyme activity under pseudo-first-order conditions.

1-6. Proteolytic Stability Assay with Repeated ProK Addition

HEPES buffer (100 mM, pH 7.3) solutions of β-Gal^{FITC} (0.3 μM) and $^{PJ}\beta$-Gal^{FITC} ([β-Gal^{FITC}] = 0.3 μM) were incubated at 37 °C. At 30-min intervals over a 3.5-h period, aliquots were withdrawn to measure enzymatic activity (**section 1-5**). Immediately following each withdrawal, Proteinase K (ProK) was added to each solution in increments of 0.3 μM. For each sample, the residual activity was expressed as a percentage of its own initial activity.

1-7. Confocal Laser Scanning Microscopy (CLSM)

HeLa cells, seeded in a 35 mm glass-bottom dish at 5.0 × 10^{4} cells dish^{-1}, were incubated at 37 °C for 24 h in DMEM containing 10% FBS in the presence of either β-Gal^{FITC} (0.1 mg mL^{-1}) or $^{PJ}\beta$-Gal^{FITC} (0.1 mg mL^{-1}). After rinsing three times with D-PBS, the cells were

incubated at 37 °C for 30 min in fresh DMEM containing 10% FBS, Hoechst 33342 (1 μM), and LysoTracker Red (30 nM). Then, the cells were rinsed three times with D-PBS and subjected to CLSM (λ_{ex} = 405 nm, 488 nm, and 561 nm). Similarly, 4T1 cells (1.0 × 10^4 cells $dish^{-1}$) were treated with $^{PJ}\beta$-Gal^{FITC} (0.1 mg mL^{-1}) and analyzed using the same staining and imaging procedures.

1-8. Flow Cytometry

Cellular Uptake Quantification. HeLa cells, seeded in a 24-well plate at 5.0 × 10^4 cells $well^{-1}$, were incubated for 24 h at 37 °C with either β-Gal^{FITC} (0.1 mg mL^{-1}) or $^{PJ}\beta$-Gal^{FITC} (0.1 mg mL^{-1}) in DMEM containing 10% FBS. After incubation, the cells were rinsed three times with D-PBS, detached from the dishes using trypsin-EDTA, and analyzed by flow cytometry (λ_{ex} = 488 nm).

Analysis of Cellular Uptake Mechanism.[57] HeLa cells, seeded in a 24-well plate at 5.0 × 10^4 cells $well^{-1}$, were subjected to one of the following four treatments. (i) Control: Cells were pre-incubated for 1 h at 37 °C in fresh DMEM containing 10% FBS, after which $^{PJ}\beta$-Gal^{FITC} (0.1 mg mL^{-1}) was added for a further 3-h incubation. (ii) Low temperature: Cells were pre-incubated for 1 h at 4 °C in fresh DMEM containing 10% FBS, after which $^{PJ}\beta$-Gal^{FITC} (0.1 mg mL^{-1}) was added for a further 3-h incubation at 4 °C. (iii) Sodium azide: Cells were pre-incubated for 1 h at 37 °C in DMEM containing 10% FBS and sodium azide (NaN_3, 10 mM), after which $^{PJ}\beta$-Gal^{FITC} (0.1 mg mL^{-1}) was added for a further 3-h incubation. (iv) Hypertonic sucrose: Cells were pre-incubated for 1 h at 37 °C in DMEM containing 10% FBS and hypertonic sucrose (400 mM), after which $^{PJ}\beta$-Gal^{FITC} (0.1 mg mL^{-1}) was added for a further 3-h incubation. After these treatments, all cells were rinsed three times with D-PBS, detached from the dishes using trypsin-EDTA, and analyzed by flow cytometry (λ_{ex} = 488 nm).

Inhibition of Cellular Uptake by TPP. HeLa cells, seeded in a 24-well plate at 5.0 × 10^4 cells $well^{-1}$, were incubated for 24 h at 37 °C in DMEM containing 10% FBS and $^{PJ}\beta$-Gal^{FITC} (0.1 mg mL^{-1}) supplemented with sodium tripolyphosphate (TPP) at concentrations of 0, 1, or 10 mM. After incubation, the cells were rinsed three times with D-PBS, detached using trypsin-EDTA, and analyzed by flow cytometry (λ_{ex} = 488 nm).

1-9. X-Gal Assay

HeLa cells, seeded in a 24-well plate at 5.0 × 10^4 cells $well^{-1}$, were incubated at 37 °C for 24 h in DMEM containing 10% FBS in the presence of either *β*-Gal^{FITC} (0.25 mg mL^{-1}) or ^{PJ}*β*-Gal^{FITC} (0.25 mg mL^{-1}). After incubation, the cells were stained using an In Situ *β*-galactosidase Staining Kit, which employs 5-bromo-4-chloro-3-indolyl-*β*-D-galactopyranoside (X-Gal) as a substrate, according to the protocol provided by the supplier. Following staining, the cells were rinsed with D-PBS and observed by bright-field optical microscopy. The percentage of blue-stained (X-Gal-positive) cells was quantified from three randomly selected bright-field images per well using ImageJ software.

2. Synthesis and Characterization

2-1. Synthesis of BP-SH

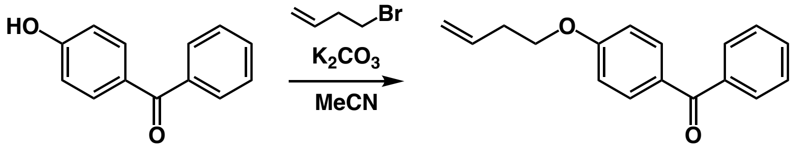

1

Compound 1. To an acetonitrile (70 mL) solution of 4-hydroxybenzophenone (6.00 g, 30.3 mmol) was added potassium carbonate (K_2CO_3, 8.28 g, 59.9 mmol). After being stirred for 10 min at room temperature, 4-bromobut-1-ene (3.5 mL, 34 mmol) was added dropwise to the mixture. The reaction mixture was refluxed for 3 h. Then, the mixture was cooled to room temperature and filtered off from an insoluble fraction. The filtrate was evaporated to dryness under reduced pressure, and the residue was extracted with AcOEt (70 mL × 2). The combined organic layers were washed with brine (50 mL), dried over anhydrous Na_2SO_4, and evaporated to dryness under reduced pressure. The residue was chromatographed on silica gel with hexane/AcOEt (95/5 to 90/10) as an eluent to afford **compound 1** as a yellow oil (5.77 g, 76%). ^{1}H NMR (500 MHz, $CDCl_3$): δ (ppm) 7.93–7.70 (m, 4H; Ar-H), 7.67–7.39 (m, 3H; Ar-H), 6.98 (d, J = 8.8 Hz, 2H; Ar-H), 6.07–5.81 (m, 1H; CH=CH_2), 5.34–5.06 (m, 2H; CH=CH_2), 4.12 (t, J = 6.7 Hz, 2H; OCH_2), 2.61 (d, J = 6.7 Hz, 2H; OCH_2CH_2). ^{13}C NMR (101 MHz, $CDCl_3$): δ (ppm) 195.61, 162.60, 138.32, 134.03, 132.58, 131.89, 130.12, 129.74, 128.20, 117.40, 114.07, 76.71, 67.44, 33.48.

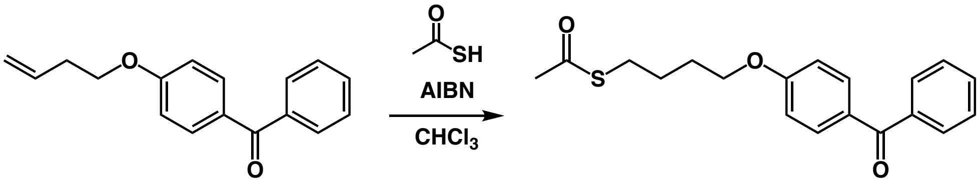

1 2

Compound 2. To a $CHCl_3$ (30 mL) solution of **1** (4.46 g, 17.7 mmol) were added thioacetic acid (1.35 g, 17.7 mmol) and 2,2'-azobis(2-methylpropanenitrile) (AIBN, 232 mg, 1.41 mmol) under Ar, and the mixture was refluxed for 12 h. The reaction mixture was evaporated to dryness under reduced pressure. The residue was dissolved in AcOEt (50 mL) and washed successively

with saturated aqueous $NaHCO_3$ (30 mL) solution and brine (30 mL). The organic layer was dried over anhydrous Na_2SO_4, filtered off from an insoluble fraction, and evaporated to dryness under reduced pressure. The residue was chromatographed on silica gel with hexane/AcOEt (95/5) as an eluent to afford **compound 2** as a yellow oil (3.57 g, 61%). ^{1}H NMR (400 MHz, $CDCl_3$): δ (ppm) 7.96 (d, J = 8.7 Hz, 2H, Ar-H), 7.91 (d, J = 8.7 Hz, 2H, Ar-H), 7.66 (t, J = 7.6 Hz, 1H, Ar-H), 7.50 (t, J = 7.6 Hz 2H, Ar-H), 7.49 (t, J = 7.6 Hz, 2H, Ar-H), 4.11 (t, J = 6.5 Hz, 2H, OCH_2), 3.04 (t, J = 7.2 Hz, 2H, SCH_2), 1.74–1.66 (m, 4H, CH_2CH_2), 2.41 (s, 3H, CH_3).^{13}C NMR (101 MHz, $CDCl_3$): δ (ppm). ^{13}C NMR (101 MHz, $CDCl_3$): δ (ppm) 195.59, 162.62, 138.33, 132.59, 131.88, 130.11, 129.74, 128.19, 114.01, 76.71, 67.51, 30.67, 28.71, 28.18, 26.27.

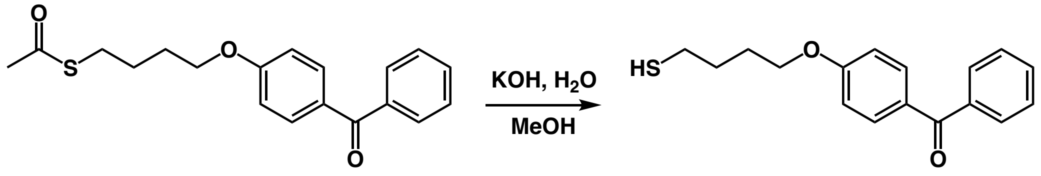

2

BP-SH

BP-SH. To a MeOH (40 mL) solution of **2** (2.00 g, 6.09 mmol) was dropwise added an aqueous (10 mL) solution of potassium hydroxide (KOH, 1.95 g, 34.8 mmol) at 0 °C under Ar. The reaction mixture was allowed to warm to room temperature and stirred for 12 h. The mixture was evaporated to dryness under reduced pressure, and the residue was extracted with AcOEt (50 mL × 2). The combined organic layer was washed with brine (30 mL), dried over anhydrous Na_2SO_4, filtered off from an insoluble fraction, and evaporated to dryness under reduced pressure. The residue was chromatographed on silica gel with hexane/AcOEt (90/10) as an eluent to afford **BP-SH** as a white solid (1.40 g, 80%). ^{1}H NMR (400 MHz, $CDCl_3$): δ (ppm) 7.84 (d, J = 8.8 Hz, 4H; Ar-H), 7.64–7.55 (m, 3H; Ar-H), 6.97 (d, J = 8.8 Hz, 2H; Ar-H), 4.09 (t, J = 6.1 Hz, 2H; OCH_2), 2.65 (d, J = 7.4 Hz, 2H; CH_2SH), 1.92–1.79 (m, 4H; CH_2CH_2), 1.42 (t, J = 7.8 Hz, 1H; CH_2SH). ^{13}C NMR (101 MHz, $CDCl_3$): δ (ppm) 162.62, 138.28, 132.60, 131.91, 130.12, 129.74, 128.20, 114.00, 76.72, 67.62, 67.57, 60.42, 38.53, 30.52, 28.33, 27.88, 27.84, 25.72, 24.38, 21.08, 14.21. ESI-MS: m/z found: 287.11 ([M + H$^+$], calcd: 287.11), 309.10 ([M + Na$^+$], calcd: 309.09).

2-2. Synthesis of ^{Gu}CD

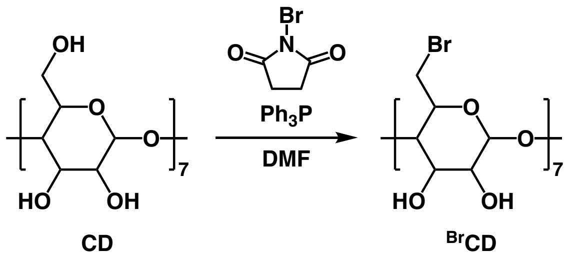

Heptakis(6-bromo-6-deoxy)-*β*-cyclodextrin (^{Br}CD).[32] To an anhydrous DMF (60 mL) solution of triphenylphosphine (Ph_3P, 32.4 g, 124 mmol) was added an anhydrous DMF (20 mL) solution of *N*-bromosuccinimide (NBS, 22.0 g, 124 mmol) at 0 °C under Ar. The mixture was warmed to room temperature and stirred for 30 min. The mixture was added dropwise to an anhydrous DMF (60 mL) solution of *β*-cyclodextrin (CD, 10.0 g, 8.81 mmol) at room temperature under Ar. The mixture was stirred at 80 °C for 12 h. After being cooled to room temperature, MeOH (10 mL) was added to the reaction mixture, and the mixture was stirred for 30 min. The mixture was then cooled to 0 °C, and the pH was adjusted to 9 with sodium methoxide. The resulting suspension was stirred for 1 h and then poured into ice-cold water (2 L). The precipitate was collected by filtration, washed with MeOH, and dried under vacuum to afford **^{Br}CD** as a white solid (9.30 g, 67%). ^{1}H NMR (400 MHz, DMSO-d_6): δ (ppm) 5.95 (d, 14H; OH-2, OH-3), 4.97 (s, 7H; H-1), 4.06 (d, 14H; H-6a, H-5), 3.73 (d, 14H; H-3, H-6b), 3.16 (s, 14H; H-2, H-4). ^{13}C NMR (101 MHz, DMSO-d_6): δ (ppm) 102.57, 85.08, 72.75, 72.50, 71.48, 49.07, 34.88.

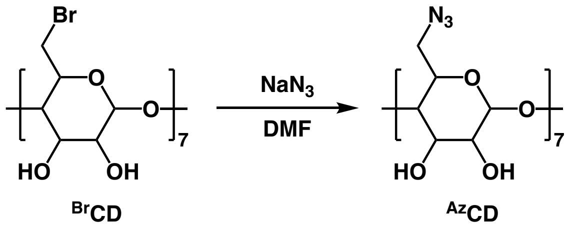

Heptakis(6-azido-6-deoxy)-*β*-cyclodextrin (^{Az}CD).[33] To an anhydrous DMF (60 mL) solution of **^{Br}CD** (8.00 g, 5.08 mmol) was added sodium azide (NaN_3, 4.62 g, 71.1 mmol), and the mixture was stirred at 70 °C for 24 h. The reaction mixture was evaporated to dryness under reduced pressure. The residue was poured into excess water, and the resulting precipitate was

collected by filtration, washed with water, and dried under reduced pressure to afford **^{Az}CD** as a white powder (5.95 g, 89 %). ^{1}H NMR (500 MHz, DMSO-d_6): δ (ppm) 5.92 (d, J = 6.5 Hz, 7H; OH-2), 5.77 (s, 7H; OH-3), 4.92 (d, J = 3.8 Hz, 7H; H-1), 3.81–3.70 (m, 14H; H-6a, H-5), 3.64–3.60 (m, 14H; H-3, H-6b), 3.57-3.30 (m, 14H; H-2, H-4).^{13}C NMR (126 MHz, DMSO-d_6): δ (ppm) 102.52, 83.66, 73.05, 72.46, 70.80, 51.79.

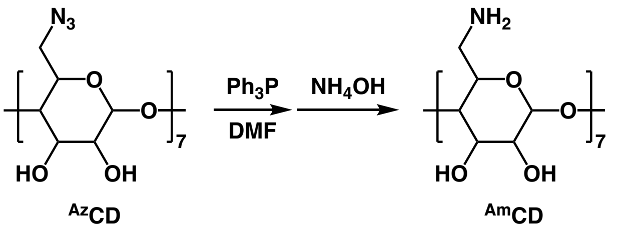

Heptakis(6-amino-6-deoxy)-*β*-cyclodextrin (^{Am}CD).[33] To an anhydrous DMF (30 mL) solution of **^{Az}CD** (4.00 g, 3.05 mmol) was added dropwise Ph_3P (5.91 g, 22.5 mmol) under Ar, and the mixture was stirred at room temperature for 12 h. To the resulting mixture, aqueous NH_4OH (28%, 9.4 mL) was added dropwise under Ar, and the mixture was stirred at room temperature for 12 h. The reaction mixture was evaporated to dryness under reduced pressure. MeOH (50 mL) was added to the residue, and the precipitate was collected by filtration, washed with MeOH, and dried under reduced pressure to afford **^{Am}CD** as a white solid (2.80 g, 81%). ^{1}H NMR (500 MHz, D_2O): δ (ppm) 5.12 (d, J = 3.6 Hz, 7H; H-1), 4.13 (m, 7H; H-5), 4.02–3.78 (m, 7H; H-3), 3.68–3.62 (m, 7H; H-2), 3.58–3.54 (m, 7H; H-4), 3.47 – 3.32 (m, 7H; H-6a), 3.27–3.11 (m, 7H; H-6b). ^{13}C NMR (126 MHz, D_2O): δ (ppm) 101.43, 82.08, 72.10, 71.56, 67.72, 66.60, 40.06.

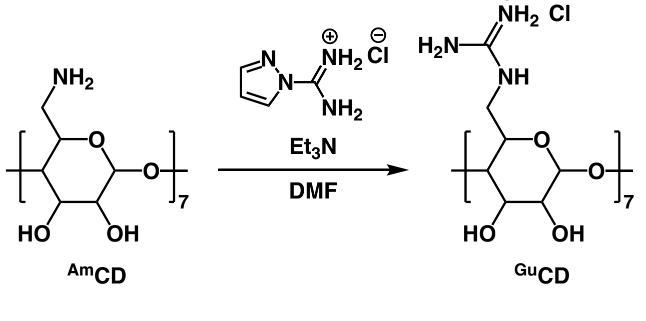

Heptakis(6-guanidino-6-deoxy)-*β*-cyclodextrin (^{Gu}CD).[33] To an anhydrous DMF (24.4 mL) solution of **^{Am}CD** (2.00 g, 1.77 mmol) and triethylamine (Et_3N, 5.84 g, 57.7 mmol) was added an anhydrous DMF (6.1 mL) solution of 1*H*-pyrazolecarboxamidine hydrochloride (4.58 g, 31.2 mmol) at 0 °C under Ar. Then, the mixture was stirred at room temperature for 2 h and subsequently at 80 °C for 12 h. After being cooled to room temperature, MeOH (3.1 mL) was added, and the resulting mixture was stirred for 30 min. The mixture was then cooled to –5 °C, and the pH was adjusted to 9 with sodium methoxide. The resulting suspension was stirred for 1 h and then poured into ice-cold water (180 mL). The precipitate was collected by filtration, washed with MeOH, and dried under vacuum to afford **^{Gu}CD** as a white solid (2.03 g, 68%). ^{1}H NMR (400 MHz, D_2O): δ (ppm) 5.21–4.87 (m, 7H; H-1), 3.99–3.87 (m, 14H; H-3, H-2), 3.69–3.56 (m, 14H; H-6a, H-8), 3.53–3.36 (m, 14H; H-4, H-6b). ^{13}C NMR (126 MHz, D_2O): δ (ppm) 158.20, 102.27, 72.93, 72.21, 71.36, 57.88, 42.73, 17.28. ESI-MS: *m*/*z* found: 765.53 ([M – 4HCl + $2H^+$], calcd: 765.79), 493.86 ([M – 6HCl + $2H^+$ + Na^+], calcd: 494.20).

2-3. ¹H and ¹³C NMR Spectroscopy

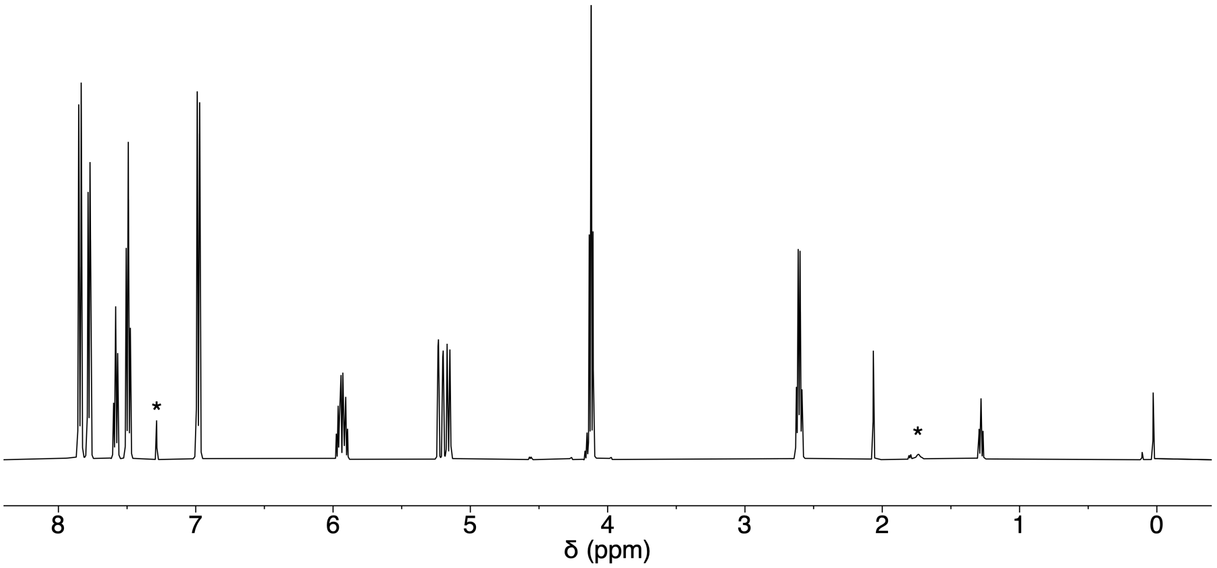

Figure S1. ^{1}H NMR spectrum of **1** in $CDCl_3$ at 25 °C. The signals marked with asterisks are due to residual solvents: $CHCl_3$ (*δ* 7.26 ppm) and H_2O (*δ* 1.74 ppm).

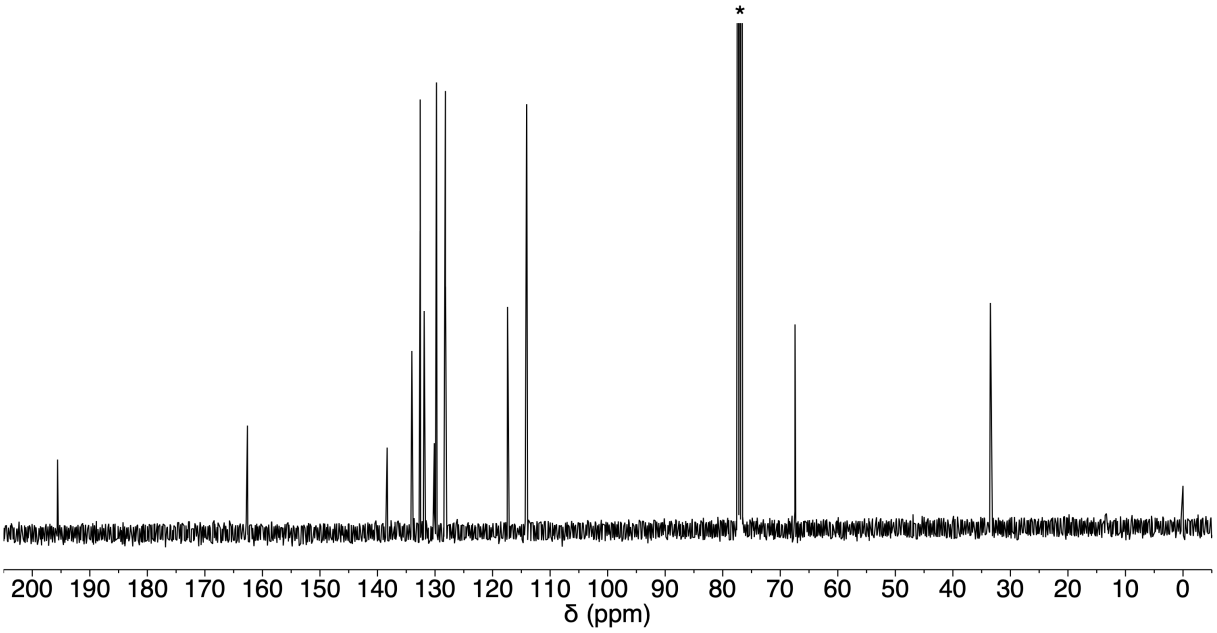

Figure S2. ^{13}C NMR spectrum of **1** in $CDCl_3$ at 25 °C. The signal marked with an asterisk is due to $CDCl_3$ (*δ* 77.3 ppm).

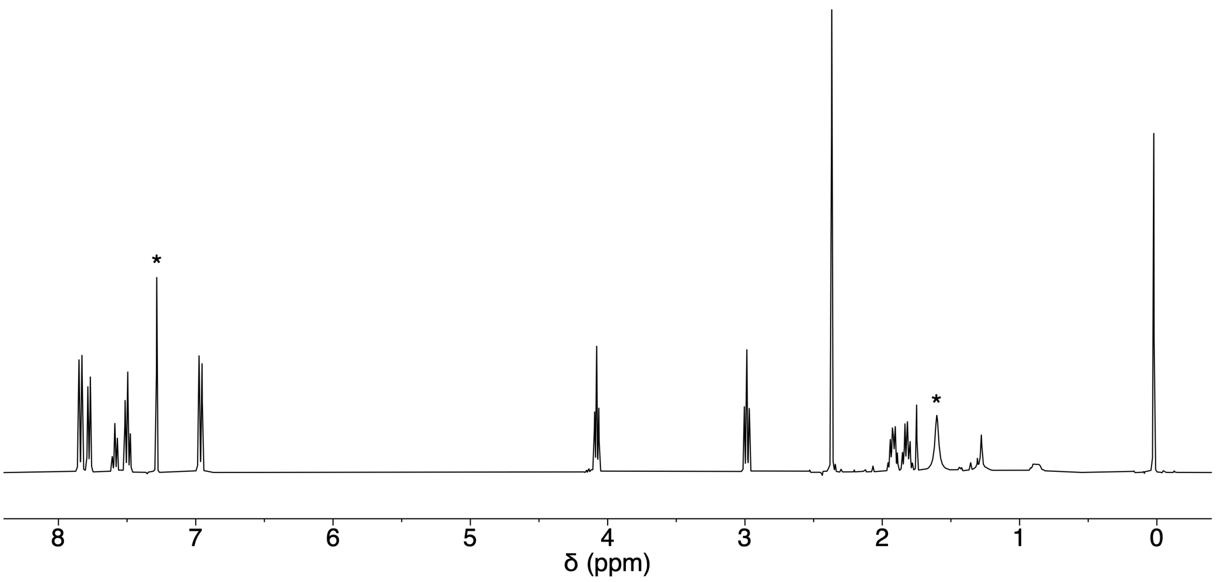

Figure S3. ^{1}H NMR spectrum of **2** in $CDCl_3$ at 25 °C. The signals marked with asterisks are due to residual solvents: $CHCl_3$ (δ 7.26 ppm) and H_2O (δ 1.60 ppm).

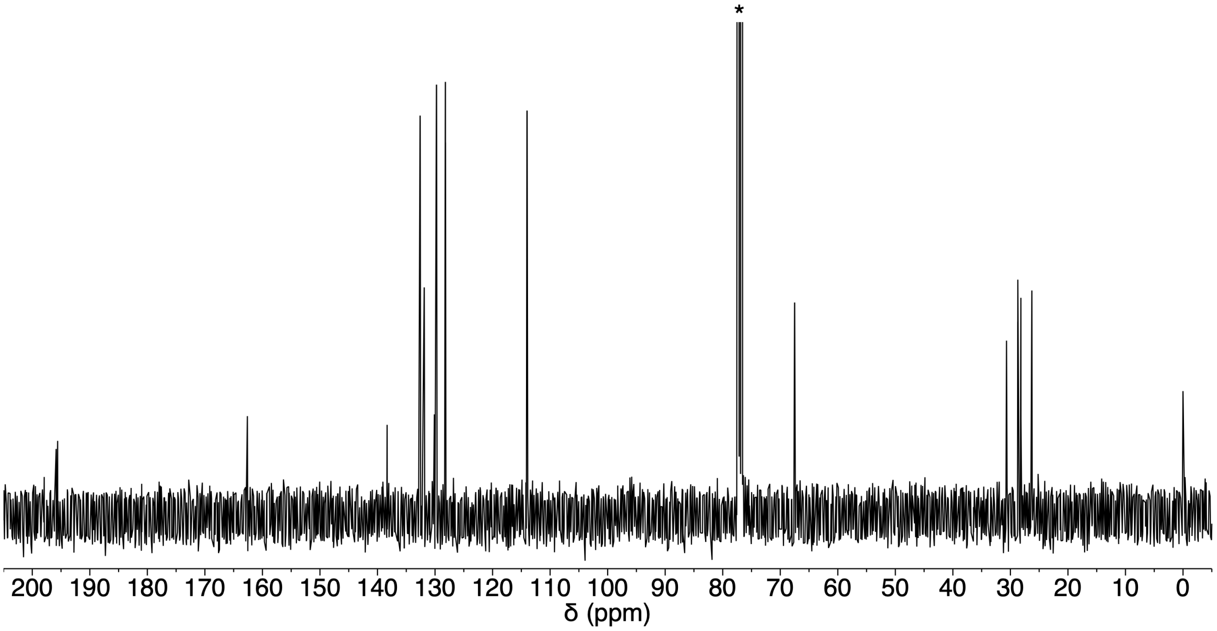

Figure S4. ^{13}C NMR spectrum of **2** in $CDCl_3$ at 25 °C. The signal marked with an asterisk is due to $CDCl_3$ (δ 77.3 ppm).

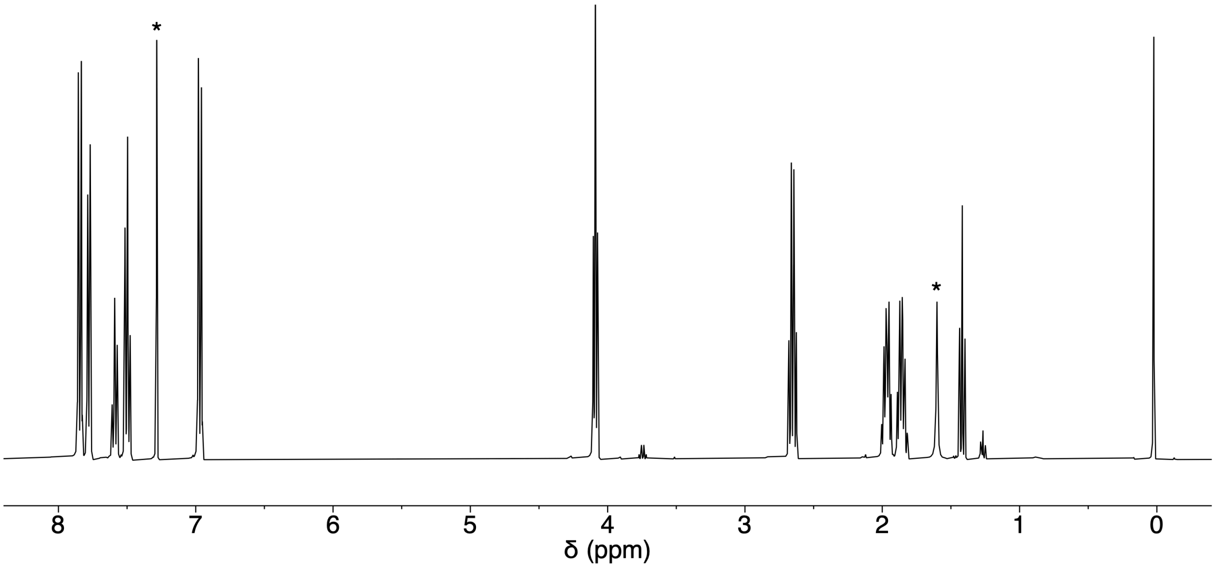

Figure S5. ^{1}H NMR spectrum of **BP-SH** in $CDCl_3$ at 25 °C. The signals marked with asterisks are due to residual solvents: $CHCl_3$ (δ 7.26 ppm) and H_2O (δ 1.60 ppm).

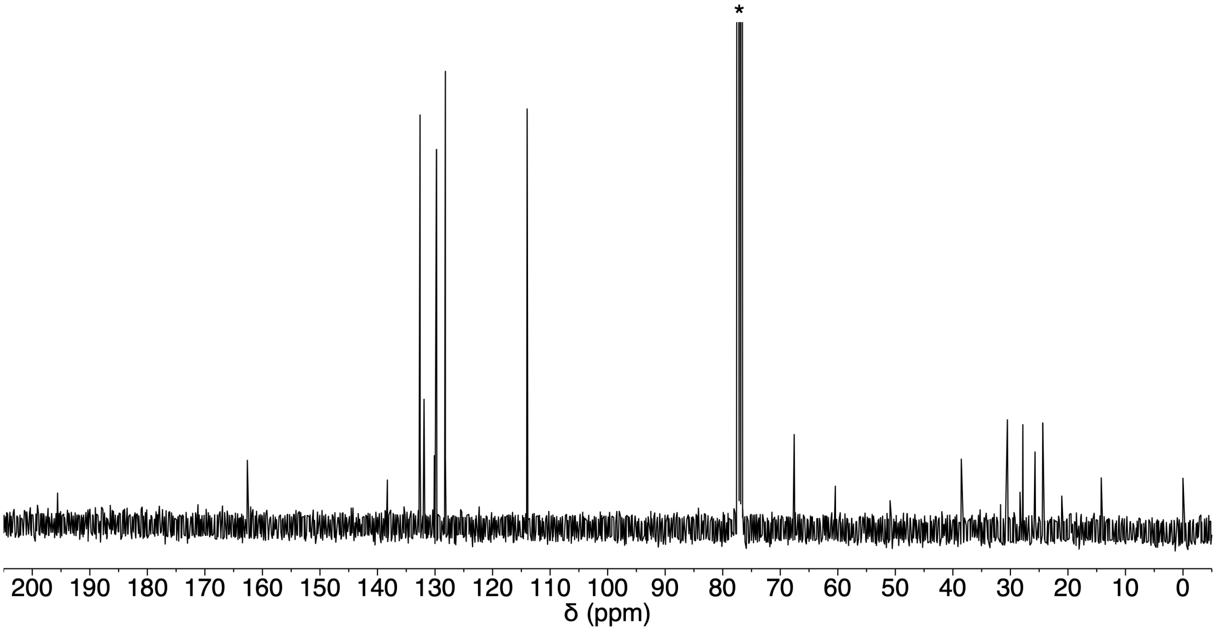

Figure S6. ^{13}C NMR spectrum of **BP-SH** in $CDCl_3$ at 25 °C. The signal marked with an asterisk is due to $CDCl_3$ (δ 77.3 ppm).

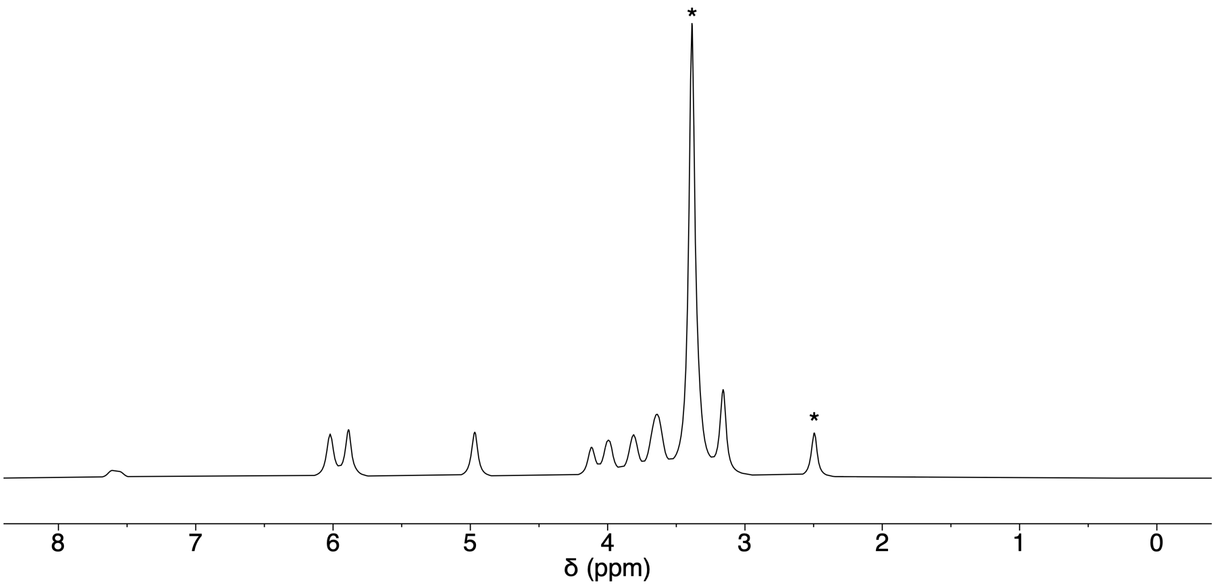

Figure S7. ^{1}H NMR spectrum of $^{\mathbf{Br}}$**CD** in DMSO-d_6 at 25 °C. The signals marked with asterisks are due to residual solvents: H_2O (δ 3.39 ppm) and DMSO-d_5 (δ 2.50 ppm).

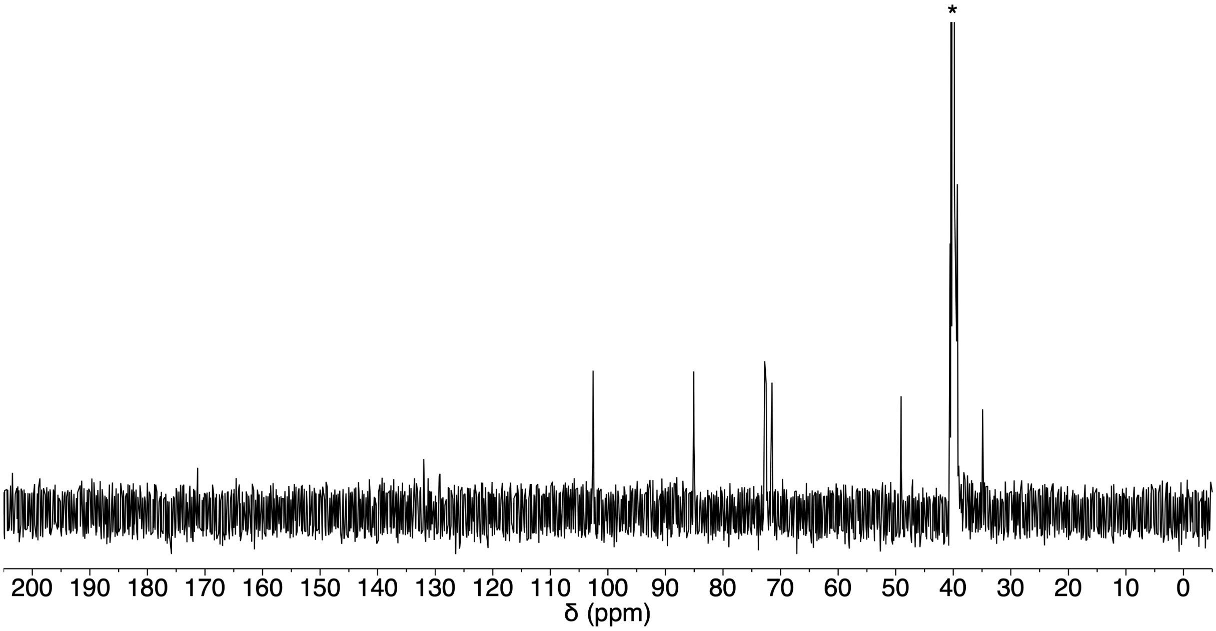

Figure S8. ^{13}C NMR spectrum of $^{\mathbf{Br}}$**CD** in DMSO-d_6 at 25 °C. The signal marked with an asterisk is due to DMSO-d_6 (δ 39.9 ppm).

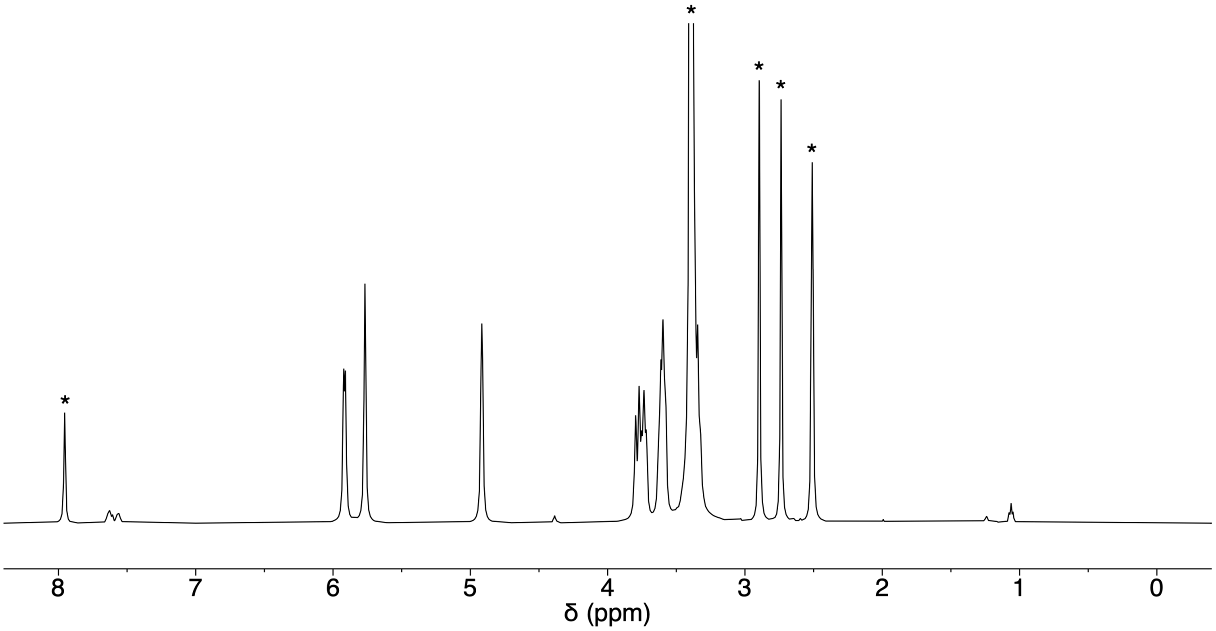

Figure S9. ^{1}H NMR spectrum of **^{Az}CD** in DMSO-d_6 at 25 °C. The signals marked with asterisks are due to residual solvents: DMF (δ 7.95, 2.90, and 2.74 ppm), H_2O (δ 3.39 ppm) and DMSO-d_5 (δ 2.51 ppm).

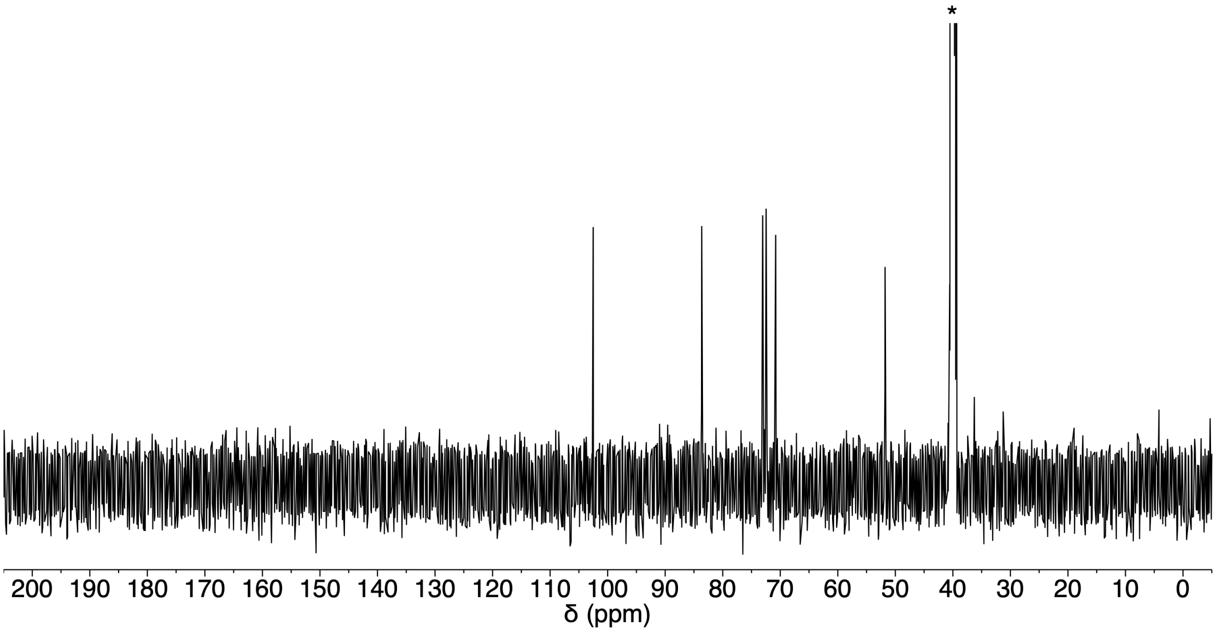

Figure S10. ^{13}C NMR spectrum of **^{Az}CD** in DMSO-d_6 at 25 °C. The signal marked with an asterisk is due to DMSO-d_6 (δ 39.9 ppm).

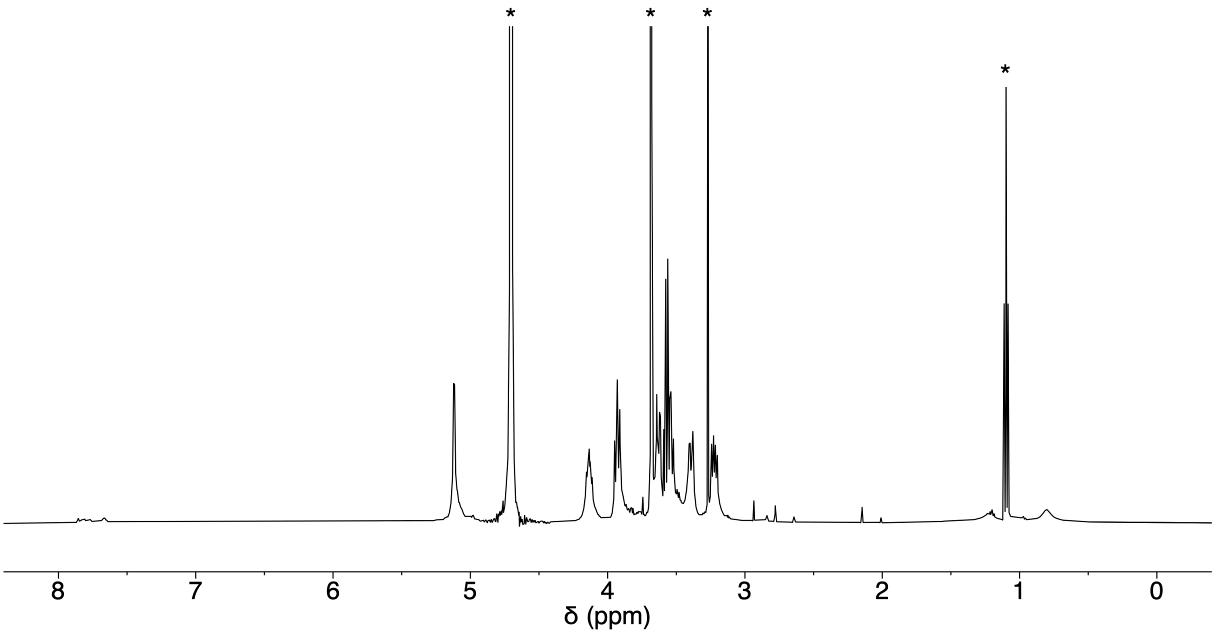

Figure S11. ^{1}H NMR spectrum of $^{\mathbf{Az}}$**CD** in D_2O at 25 °C. The signals marked with asterisks are due to residual solvents: H_2O (δ 4.70 ppm), EtOH (δ 3.70 and 1.10 ppm), and MeOH (δ 3.27 ppm).

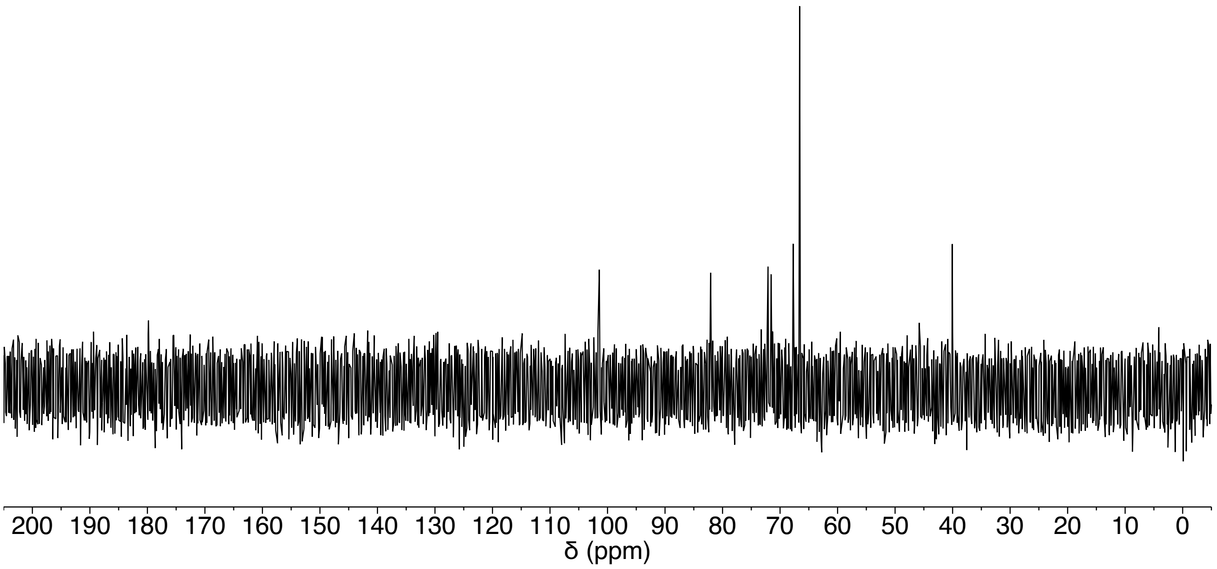

Figure S12. ^{13}C NMR spectrum of $^{\mathbf{Am}}$**CD** in D_2O at 25 °C.

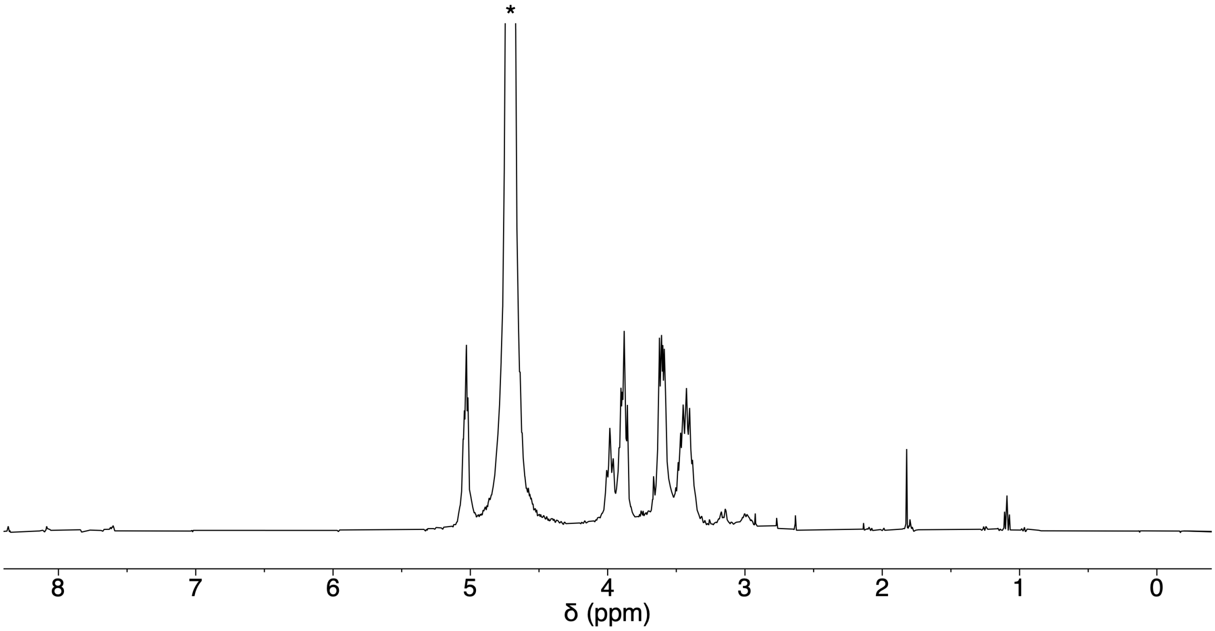

Figure S13. ^{1}H NMR spectrum of $^{\mathbf{Gu}}$**CD** in D_2O at 25 °C. The signal marked with an asterisk is due to H_2O (δ 4.70 ppm).

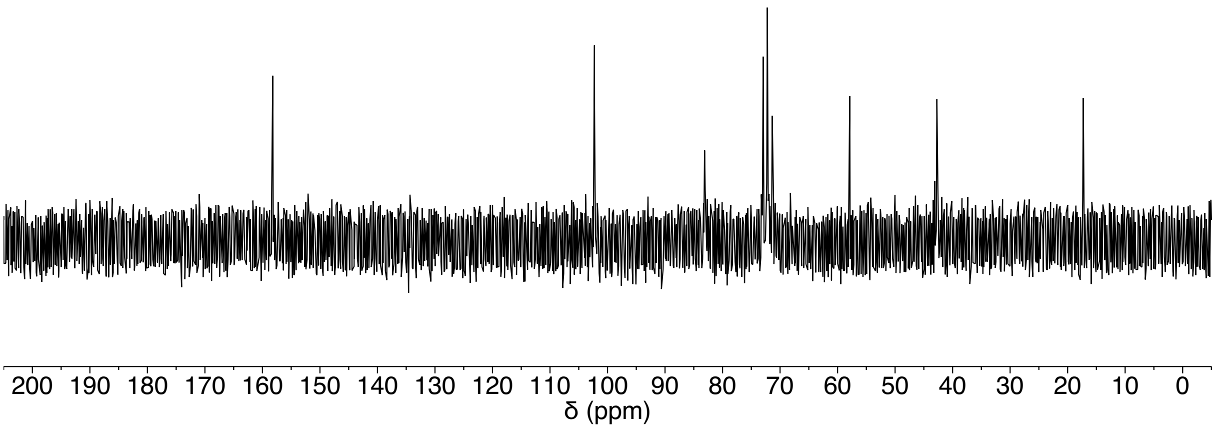

Figure S14. ^{13}C NMR spectrum of $^{\mathbf{Gu}}$**CD** in D_2O at 25 °C.

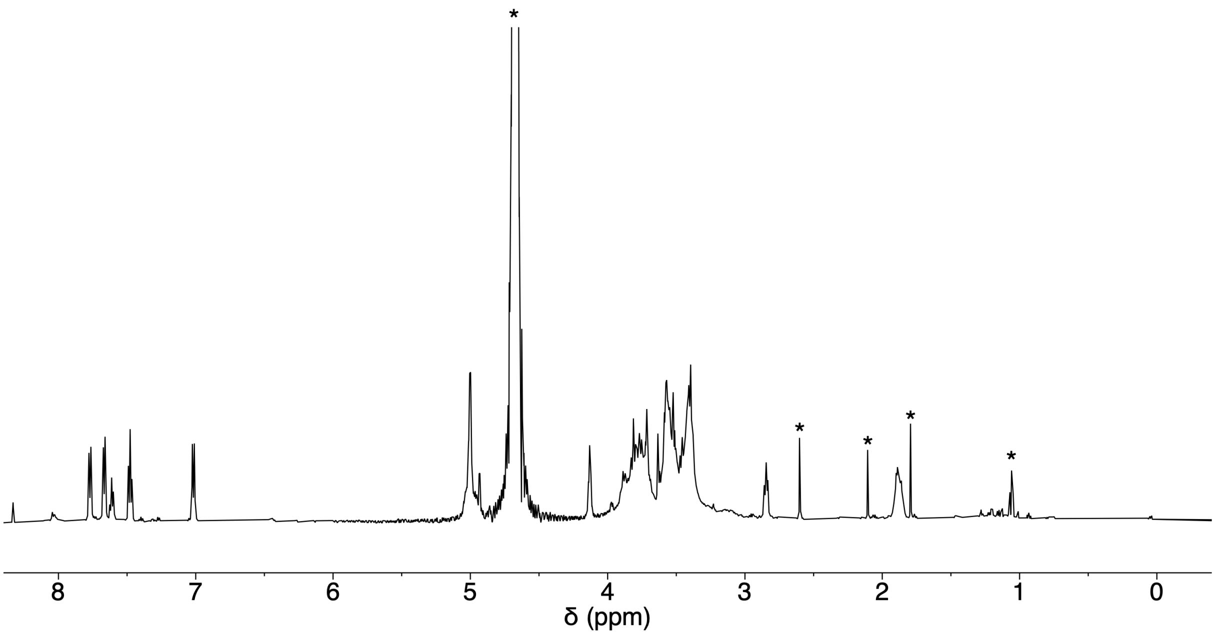

Figure S15. ^{1}H NMR spectrum of ^{Gu}**CD⊃BP-SH** in D_2O at 25 °C. The signals marked with asterisks are due to residual solvents: H_2O (*δ* 4.67 ppm), DMSO (*δ* 2.60 ppm), acetic acid/acetate ion (*δ* 2.11 and 1.79 ppm), EtOH (*δ* 1.07 ppm).

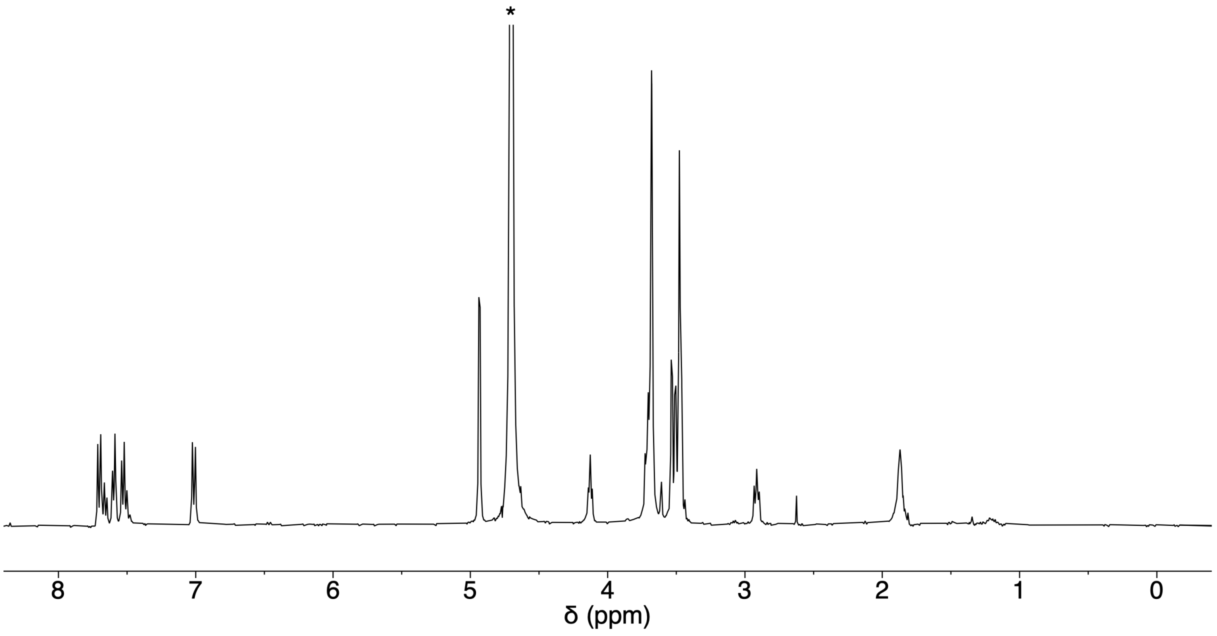

Figure S16. ^{1}H NMR spectrum of **CD⊃BP-SH** in D_2O at 25 °C. The signal marked with an asterisk is due to H_2O (*δ* 4.70 ppm).

2-4. Electronic Absorption Spectroscopy

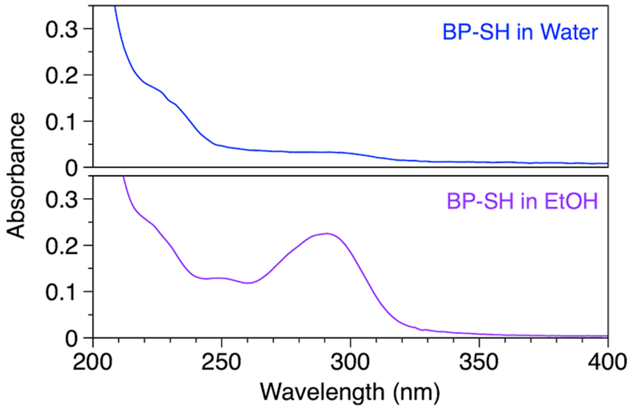

Figure S17. Absorption spectra of BP-SH (1 mg mL^{-1}) in water (blue) and EtOH (purple).

2-5. Dynamic Light Scattering (DLS) and Zeta-potential (*ζ*) Measurements

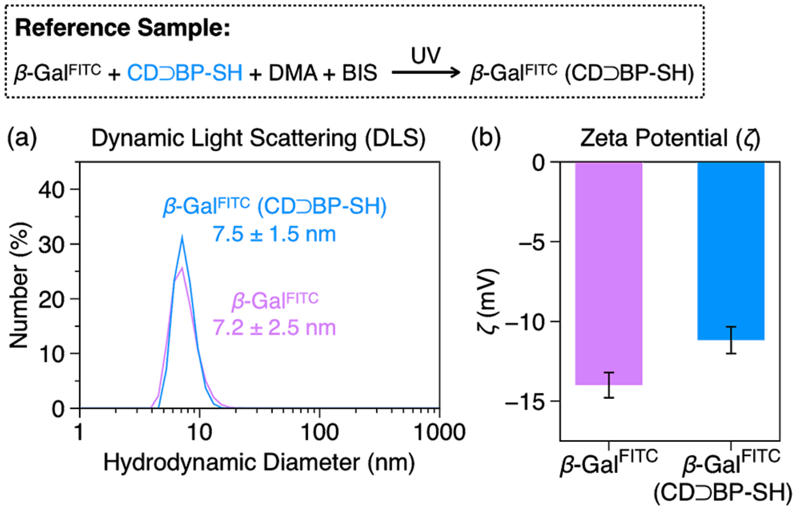

Figure S18. (a) Dynamic light scattering (DLS) profiles and (b) zeta potentials of *β*-Gal^{FITC} (5 mg mL^{-1}) and *β*-Gal^{FITC} (CD⊃BP-SH) (5 mg mL^{-1}), measured in HEPES buffer (100 mM, pH 7.3).

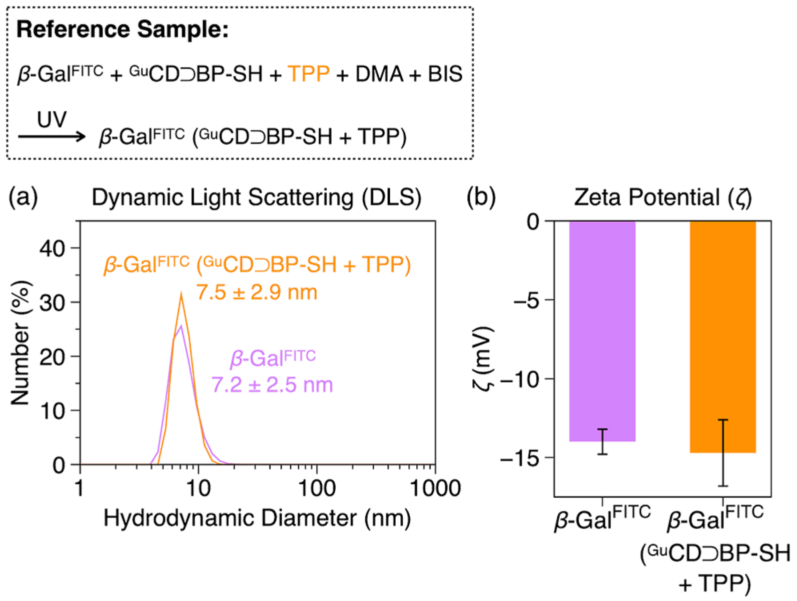

Figure S19. (a) Dynamic light scattering (DLS) profiles and (b) zeta potentials of β-Gal^{FITC} (5 mg mL^{-1}) and the reference sample β-Gal^{FITC} (^{Gu}CD⊃BP-SH + TPP), prepared in the presence of sodium tripolyphosphate (TPP). Both samples were measured at 5 mg mL^{-1} in HEPES buffer (100 mM, pH 7.3).

2-6. Screening of $^{PJ}\beta$-Gal^{FITC} Preparation Conditions

$^{PJ}\beta$-Gal^{FITC} samples were prepared according to the protocol described in **section 1-4** using various concentrations of β-Gal^{FITC}, ^{Gu}CD⊃BP-SH, DMA, and BIS. To evaluate protease resistance, the samples were incubated with 5 molar equivalents of ProK at 37 °C for 24 h, and the remaining activity was normalized to the initial activity measured before treatment.

Table S1. Hydrodynamic diameters (D_h) and zeta potentials (ζ) of $^{PJ}\beta$-Gal^{FITC} samples prepared under different conditions, and their residual enzymatic activity after Proteinase K (ProK) treatment.

Entry	[β-Gal^{FITC}] (μM)	[^{Gu}CD⊃BP-SH] (μM)	[DMA] (mM)	[BIS] (mM)	D_h (nm)	ζ (mV)	Residual Activity after ProK Treatment (%)
**β-Gal^{FITC}**					7.2 ± 2.5	−14	44
**1**	5	50	25	25	59 ± 14	−14.7	61
**2**	5	50	50	50	58 ± 15	−14.6	60
**3**	5	50	100	100	174 ± 38	−14	62
**4**	5	100	25	25	119 ± 26	−11.5	63
**5**	5	100	50	50	168 ± 31	−10.5	72
**6**	5	100	100	100	223 ± 47	−10.8	65
**7**	5	150	25	25	189 ± 36	−6.2	80
**8**	5	150	50	50	192 ± 42	−7.3	77
**9**	5	150	100	100	447 ± 83	−8.5	77
**10**	2	60	25	25	73 ± 18	−6.3	86
**11**	2	60	50	50	84 ± 20	−5.7	84
**12**	2	60	100	100	204 ± 37	−6.8	85

2-7. Circular Dichroism Spectroscopy

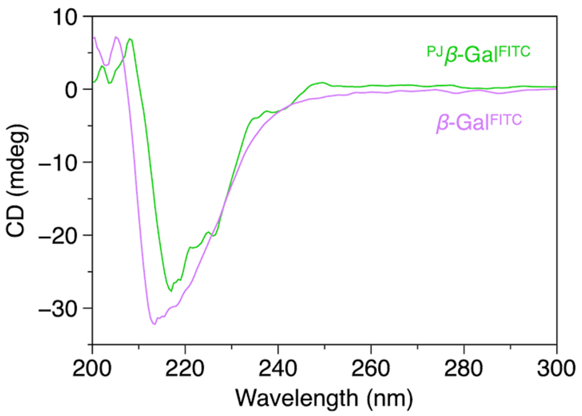

Figure S20. Circular dichroism (CD) spectra of β-Gal^{FITC} (50 μM) and $^{PJ}\beta$-Gal^{FITC} ([β-Gal^{FITC}] = 50 μM), measured in HEPES buffer (100 mM, pH 7.3).

2-8. SDS-PAGE

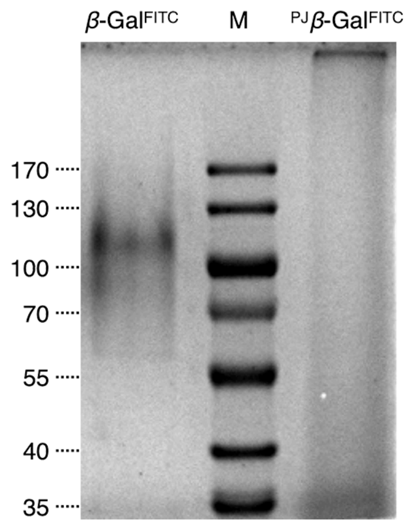

Figure S21. SDS-PAGE analysis of β-Gal^{FITC} (2.5 mg mL^{-1}) and $^{PJ}\beta$-Gal^{FITC} (10 mg mL^{-1}). The gel (8%) was stained with Coomassie Brilliant Blue. M: Molecular weight marker (values in kDa).

2-9. Kinetic Analysis of $^{PJ}\beta$-Gal^{FITC}

Table S2. Kinetic parameters of β-Gal^{FITC} and $^{PJ}\beta$-Gal^{FITC}. Data represent the best-fit values ± standard error (SE) derived from non-linear regression analysis using the Michaelis-Menten model. The 95% confidence intervals (CI) and goodness-of-fit (R^2) are also provided.

Sample	K_m (mM)	V_{max} (μM s^{-1})	k_{cat} (s^{-1})	k_{cat}/K_m (mM^{-1} s^{-1})	R^2
**β-Gal^{FITC}**					
Value ± SE	1.04 ± 0.08	2.05 ± 0.02	10.3 ± 0.1	9.88 ± 0.74	0.990
95% CI	[0.79, 1.28]	[1.98, 2.12]	[9.9, 10.6]	[7.53, 12.22]	–
**$^{PJ}\beta$-Gal^{FITC}**					
Value ± SE	2.29 ± 0.22	1.42 ± 0.03	7.1 ± 0.1	3.11 ± 0.30	0.989
95% CI	[1.60, 2.98]	[1.33, 1.51]	[6.6, 7.6]	[2.15, 4.07]	–

3. Structural Analysis of *β*-Galactosidase Surface

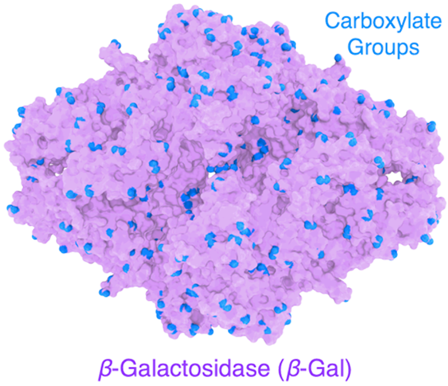

Figure S22. Surface carboxylate distribution on *β*-galactosidase (PDB ID: 1DP0). The carboxylate groups from surface-exposed aspartic and glutamic acid residues are highlighted in blue.

4. Fluorescence Spectroscopy

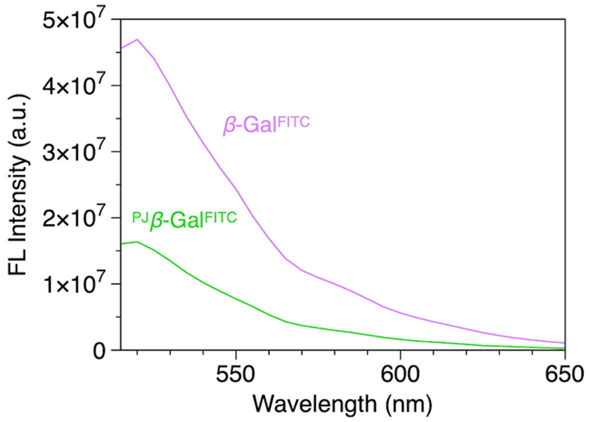

Figure S23. Fluorescence (λ_{ex} = 488 nm) spectra of β-Gal^{FITC} (0.1 mg mL^{-1}, purple) and $^{PJ}\beta$-Gal^{FITC} (0.1 mg mL^{-1}, purple) in water.

5. Nanoparticle Tracking Analysis (NTA)

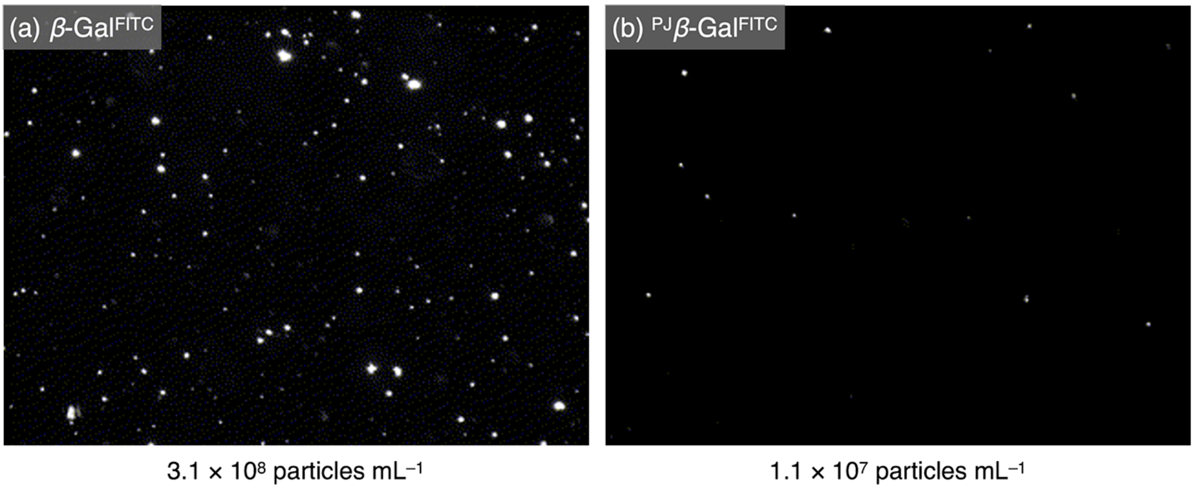

Figure S24. Nanoparticle tracking analysis (NTA) images of (a) *β*-Gal^{FITC} (0.1 mg mL^{-1}) and ^{PJ}*β*-Gal^{FITC} (0.1 mg mL^{-1}) in water.

6. Enzymatic Activity Assay

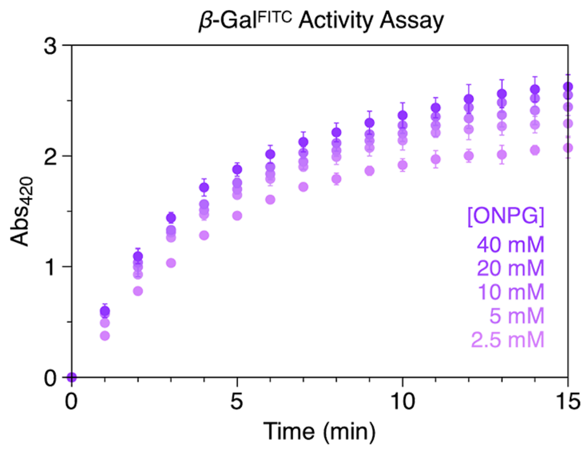

Figure S25. Changes in absorbance at 420 nm (Abs_{420}) at 37 °C for 0–15 min of HEPES buffer (100 mM, pH 7.3) containing β-Gal^{FITC} (0.2 μM) and ONPG at different concentrations (2.5–40 mM). Data are presented as the mean ± SD (n = 3).

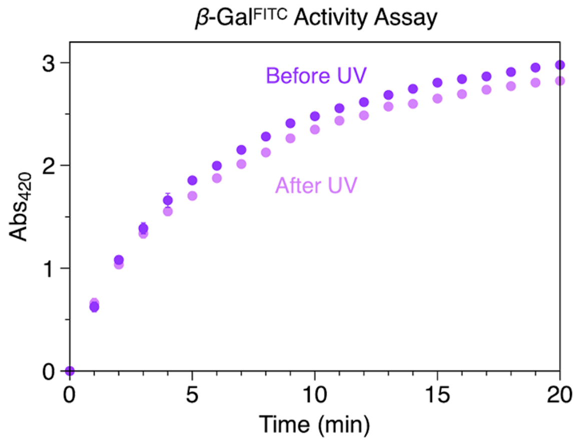

Figure S26. Changes in absorbance at 420 nm (Abs_{420}) at 37 °C for 0–20 min of HEPES buffer (100 mM, pH 7.3) containing β-Gal^{FITC} (0.2 μM) and ONPG (40 mM). Data were collected for the sample before and after UV irradiation (365 nm, 30 min). Data are presented as the mean ± SD (n = 3).

7. Cell Viability Assay

HeLa cells, seeded in a 96-well plate at 5.0×10^3 cells well^{-1}, were incubated at 37 °C for 24 h in 100 μL of DMEM containing 10% FBS and $^{PJ}\beta$-Gal^{FITC} at various concentrations (0.03125–0.75 mg mL^{-1}). Subsequently, 10 μL of Cell Counting Kit-8 (CCK-8) solution was added to each well. After a further 1-h incubation at 37 °C, the absorbance at 450 nm was measured.

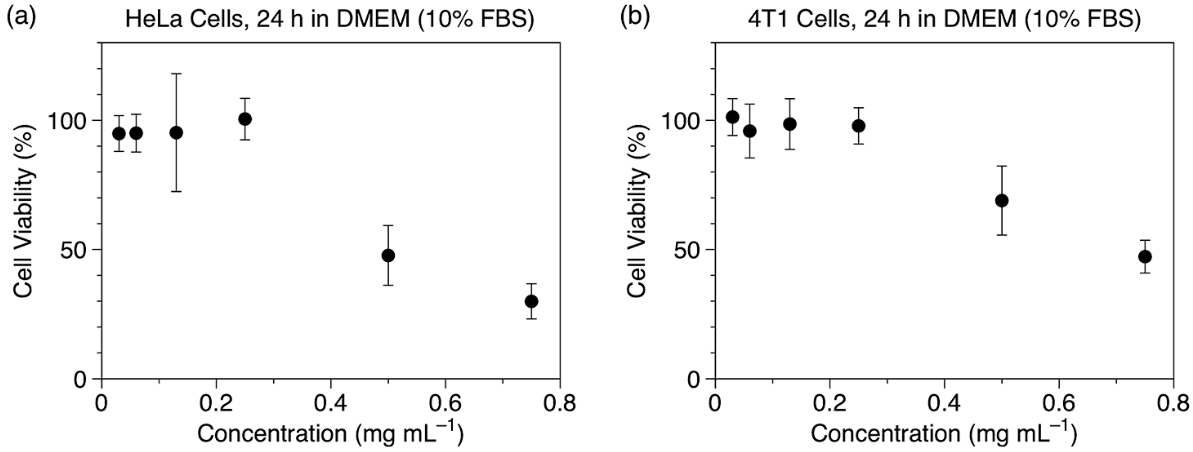

Figure S27. Viability of (a) HeLa and (b) 4T1 cells treated with $^{PJ}\beta$-Gal^{FITC} (0.03125–0.75 mg mL^{-1}) at 37 °C in DMEM containing 10% FBS for 24 h. Data are presented as the mean ± SD (n = 3).

8. Confocal Laser Scanning Microscopy (CLSM)

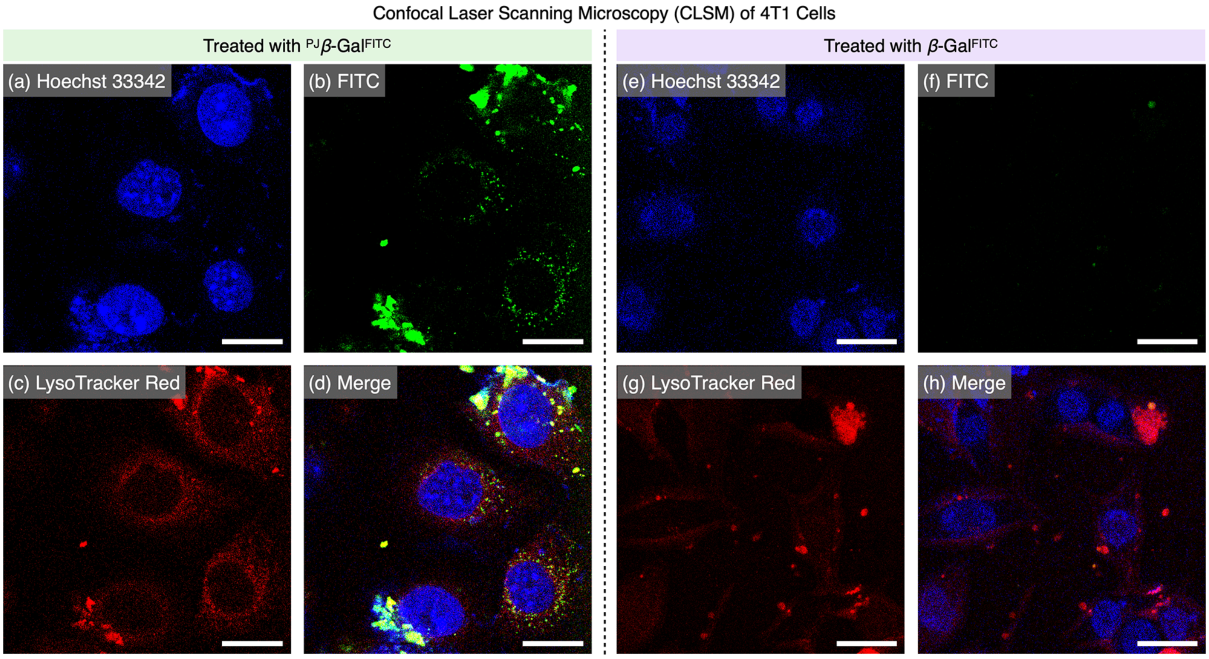

Figure S28. Confocal laser scanning microscopy (CLSM) of 4T1 cells prepared by a 24-h incubation with (a–d) $^{PJ}\beta$-Gal^{FITC} (0.1 mg mL^{-1}) or (e–h) β-Gal^{FITC} (0.1 mg mL^{-1}), followed by staining with Hoechst 33342 and LysoTracker Red. Images display fluorescence from (a, e) Hoechst 33342 (blue; λ_{ex} = 405 nm), (b, f) FITC (green; λ_{ex} = 488 nm), (c, g) LysoTracker Red (red; λ_{ex} = 561 nm), and (d, h) their merged view. Scale bars: 25 μm.

9. Flow Cytometry

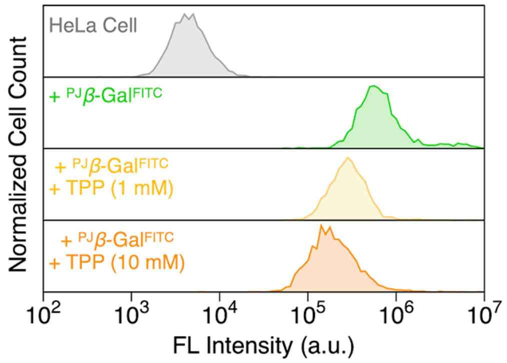

Figure S29. Flow cytometry histograms (λ_{ex} = 488 nm) of HeLa cells before and after a 24-h incubation with ${}^{PJ}\beta$-Gal^{FITC} (0.1 mg mL^{-1}) in the absence or presence of sodium tripolyphosphate (TPP; 1 mM or 10 mM).
